# B cells in perivascular and peribronchiolar granuloma-associated lymphoid tissue and B-cell signatures identify asymptomatic *Mycobacterium tuberculosis* lung infection in Diversity Outbred mice

**DOI:** 10.1128/iai.00263-23

**Published:** 2024-06-20

**Authors:** Deniz Koyuncu, Thomas Tavolara, Daniel M. Gatti, Adam C. Gower, Melanie L. Ginese, Igor Kramnik, Bülent Yener, Usama Sajjad, Muhammad Khalid Khan Niazi, Metin Gurcan, Anas Alsharaydeh, Gillian Beamer

**Affiliations:** 1 Rensselaer Polytechnic Institute, Troy, New York, USA; 2 Wake Forest University, School of Medicine, Winston Salem, North Carolina, USA; 3 The Jackson Laboratory, Bar Harbor, Maine, USA; 4 Boston University Clinical and Translational Science Institute, Boston, Massachusetts, USA; 5 Tufts University Cummings School of Veterinary Medicine, North Grafton, Massachusetts, USA; 6 NIEDL, Boston University, Boston, Massachusetts, USA; 7 Aiforia Inc., Cambridge, Massachusetts, USA; 8 Texas Biomedical Research Institute, San Antonio, Texas, USA; University of Illinois Chicago, Chicago, Illinois, USA

**Keywords:** *tuberculosis*, *Mycobacterium tuberculosis*, Diversity Outbred mice, host-pathogen interactions, immunity

## Abstract

Because most humans resist *Mycobacterium tuberculosis* infection, there is a paucity of lung samples to study. To address this gap, we infected Diversity Outbred mice with *M. tuberculosis* and studied the lungs of mice in different disease states. After a low-dose aerosol infection, progressors succumbed to acute, inflammatory lung disease within 60 days, while controllers maintained asymptomatic infection for at least 60 days, and then developed chronic pulmonary tuberculosis (TB) lasting months to more than 1 year. Here, we identified features of asymptomatic *M. tuberculosis* infection by applying computational and statistical approaches to multimodal data sets. Cytokines and anti-*M*. *tuberculosis* cell wall antibodies discriminated progressors vs controllers with chronic pulmonary TB but could not classify mice with asymptomatic infection. However, a novel deep-learning neural network trained on lung granuloma images was able to accurately classify asymptomatically infected lungs vs acute pulmonary TB in progressors vs chronic pulmonary TB in controllers, and discrimination was based on perivascular and peribronchiolar lymphocytes. Because the discriminatory lesion was rich in lymphocytes and CD4 T cell-mediated immunity is required for resistance, we expected CD4 T-cell genes would be elevated in asymptomatic infection. However, the significantly different, highly expressed genes were from B-cell pathways (e.g., *Bank1*, *Cd19*, *Cd79*, *Fcmr*, *Ms4a1*, *Pax5*, and *H2-Ob*), and CD20+ B cells were enriched in the perivascular and peribronchiolar regions of mice with asymptomatic *M. tuberculosis* infection. Together, these results indicate that genetically controlled B-cell responses are important for establishing asymptomatic *M. tuberculosis* lung infection.

## INTRODUCTION

Tuberculosis (TB) is a globally important disease. Over 2 billion people harbor infection with *Mycobacterium tuberculosis*, resulting in 9–10 million new TB cases and 1.6 million deaths (1). Humans respond variably to *M. tuberculosis* infection, ranging from extreme susceptibility to extreme resistance. Only 5%–10% of infected adult humans develop lung disease, very rarely developing acute fulminant TB and commonly developing post-primary TB, a destructive lung disease that can wax and wane (2–5). Most humans are highly resistant to *M. tuberculosis* and clear bacilli by innate immunity (6) or restrict bacillary growth by adaptive immunity and maintain asymptomatic latent *M. tuberculosis* infection (7). Severe immune deficiency, malnutrition, vitamin D deficiency, diabetes, extreme age, smoking, illicit drug use, and co-infections are known risk factors for pulmonary TB. However, the presence of these risk factors does not fully explain susceptibility, and absence does not fully explain resistance (8–11).

Additional factors, such as genetically controlled immune and inflammatory responses, contribute to resistance and susceptibility to *M. tuberculosis* (12, 13). Large effects due to host genetic background have been shown in immune-competent mice by using panels of Collaborative Cross inbred mouse strains (14, 15) and by using the Diversity Outbred mouse population (16–18). We and others have shown that aerosolized *M. tuberculosis* causes severe, acute necrotizing inflammation that fails to restrict *M. tuberculosis* growth in ~30% of Diversity Outbred mice, and these progressors succumb to acute pulmonary TB within 60 days (16, 18, 19). The remaining ~70% of Diversity Outbred mice survive longer, have significantly lower levels of acute inflammation, and develop non-necrotizing lung granulomas that better restrict *M. tuberculosis* (16, 18, 20, 21).

Previously, we used multiple approaches to study progressors, including granuloma analysis, transcriptional profiling, statistical analyses, and machine learning methods to find and validate biomarkers (18, 22). We developed and validated a weakly supervised, attention-based, multiple instance learning model that automatically diagnosed progressors with high accuracy (91.50% ± 4.68%) from digital granuloma images. Furthermore, post hoc visual examination by board-certified pathologists confirmed that the imaging biomarker of progressors was human interpretable and corresponded to pyknotic nuclear debris within granulomas (23). Transcriptional analyses identified a neutrophil-associated inflammatory lung signature in progressors, which subsequently led to discovery and validation of diagnostic biomarkers for pulmonary TB, one of which (the neutrophil chemokine, serum CXCL1) met the World Health Organization’s diagnostic criteria for a triage test in human sera (22).

Here, we extend that work by showing the culmination of large survival studies lasting nearly 2 years and novel approaches to uncover features of asymptomatic resistance to *M. tuberculosis* in Diversity Outbred mice.

## MATERIALS AND METHODS

### Mice

Female Diversity Outbred mice (4–5 weeks old; *n* = 1,009) from generations 15, 16, 21, 22, 34, 35, 37, and 42 were purchased from The Jackson Laboratory (Bar Harbor, ME, USA) and group housed (*n* = 5–7 mice per cage) on Innovive (San Diego, CA, USA) or Allentown Inc (Allentown, NJ, USA) ventilated, HEPA-filtered racks in the New England Regional Biosafety Laboratory (Tufts University, Cummings School of Veterinary Medicine, North Grafton, MA, USA). The light cycle was 12 hours of light and 12 hours of dark. Disposable caging was purchased sterile, and re-usable caging was autoclaved prior to use. All cages were lined with sterile corncob bedding and sterile paper nestlets (Scotts Pharma Solutions, Marlborough, MA, USA). Cages were changed at least every other week. The mice were provided with sterile mouse chow (Envigo, Indianapolis, IA, USA) and sterile, acidified water *ad libitum*.

### *M. tuberculosis* aerosol infection

At 8–10 weeks old, the mice were infected with aerosolized *M. tuberculosis* strain Erdman using a custom-built CH Technologies system (18, 22, 24). Twenty-four hours after each aerosol run, 4–12 mice were euthanized by carbon dioxide; the entire lungs were homogenized in 5-mL sterile phosphate-buffered saline; and all homogenates were plated onto oleic albumin dextrose catalase (OADC)-supplemented 7H11 agar. After 3 weeks at 37°C, *M. tuberculosis* colony-forming units were counted to determine the retained lung dose (18, 22, 24). The mice were infected with ~100 bacilli in the first two experiments and ~25 bacilli in the subsequent eight experiments.

### Clinical monitoring and survival

The mice were observed daily for routine health monitoring. The mice were weighed before *M. tuberculosis* aerosol infection and at least once per week afterward until consecutive weight loss was noted, and then were weighed up to daily. Criteria requiring euthanasia were any one of the following: severe weakness/lethargy, respiratory difficulty, or body condition score of 2 (25). We confirmed morbidity was due to pulmonary TB by finding (i) large nodular or severe diffuse lung lesions; (ii) histopathological confirmation of neutrophilic, lymphoplasmacytic, histiocytic, or granulomatous lung infiltrates; (iii) cultivable *M. tuberculosis* bacilli from lung tissue; and (iv) absence of other disease based on necropsy findings. Since the Institutional Animal Care and Use Committee disallowed natural death as an endpoint, the day a mouse was euthanized due to morbidity was used as a proxy of survival. Mice with morbidity not attributable to pulmonary TB were excluded from subsequent analyses. Progressors succumbed to pulmonary TB and were euthanized due to morbidity prior to 60 days post-infection. Controllers succumbed to pulmonary TB and were euthanized due to morbidity after 60 days post-infection. Asymptomatically infected Diversity Outbred mice were euthanized at a predetermined timepoint (on or before 60 days), and their classification state was determined by normal behaviors, postures, movements, eating/drinking, respiration, and weight gain.

### Quantification of *M. tuberculosis* lung burden

Immediately after euthanasia, two or three lung lobes were removed from each mouse and homogenized in 1-mL sterile phosphate-buffered saline per lobe, serially diluted, plated onto OADC-supplemented 7H11 agar, and incubated at 37°C. After 3–4 weeks, *M. tuberculosis* colonies were counted, and *M. tuberculosis* burden in the lungs was calculated (18, 22, 24).

### Histology

One or two lung lobes from each Diversity Outbred mouse were inflated and fixed in 10% neutral buffered formalin, processed, embedded in paraffin, sectioned at 5 micron, and stained with carbol fuschin for acid-fast bacteria followed by counterstaining with hematoxylin and eosin (H&E) at the Cummings School of Veterinary Medicine’s Comparative Genomics and Pathology Shared Resource (North Grafton, MA, USA). Stained tissue sections on glass slides were digitally scanned by Aperio ScanScope or AT2 scanners at 0.23 microns/pixel at Vanderbilt University Medical Center’s Digital Histology Shared Resource (Nashville, TN, USA). A separate set of tissue sections from Diversity Outbred mice was stained using immunohistochemistry to detect CD20 on B cells. Slides were digitally scanned by an Olympus VS2000 scanner at 0.138 microns/pixel at The Ohio State University’s Comparative Pathology and Digital Imaging Shared Resource (Columbus, OH, USA).

### Deep-learning and image analysis on lung tissue dual-stained by carbol fuschin and H&E

Lung tissue section images from 129 *M*. *tuberculosis-*infected Diversity Outbred mice were used for training, and 98 different images were used as a hold-out test set. Of the 129 training images, 66 were from asymptomatic mice and 63 images were from controllers. Of the 98 test set images, 10 were asymptomatic mice and 88 were controllers. Following image preprocessing (see Supplemental Methods), we used an attention-based multiple instance learning method (26) to identify regions of the images that contributed to classification and yielded an interpretable model (23). Briefly, we assigned lung tissue images based on the host classes (controllers and asymptomatic) and trained an end-to-end deep-learning model to predict the image-level class. The model consisted of two parts: a feature extractor followed by the attention mechanism, depicted in Fig. S5. Optimization used the Adam optimizer with the following parameters: *β*1 = 0.9 and *β*2 = 0.999, an learning rate of 0.0001, a weight decay of 0.0005, and over 100 epochs. For each fold, 30 Diversity Outbred mice were randomly sampled for the validation set, and the rest of the cases were used for the training of the model, known as Monte Carlo cross-validation (27). To account for imbalanced training sets, the training procedure was modified to randomly select images from controller or asymptomatic mice with equal likelihood and then to randomly select a mouse from the selected category during every training iteration. Negative log-likelihood was used as a cost function.

### Deep-learning and image analysis on lung sections stained by immunohistochemistry for CD20

Peribronchiolar and perivascular regions were segmented based on a board-certified veterinary pathologist’s (G.B.) manual training annotations using Aiforia Create (v.6.0) with default parameter settings (Aiforia Technologies, Helsinki, Finland). To quantify CD20+ B cells within the peribronchiolar and perivascular regions, we first identified the nuclei within the segmented regions at ×40 magnification using CellViT-SAM-H-x40 (28). Next, to identify CD20+ (brown colored) pixels, we used an entropy-based cell quantification method (29). Entropy-based cell quantification makes use of the uniform and perceptually aligned color representation of International Commission on Illumination (CIE) L*a*b* color space (30) to transform the complex quantification problem into an automatic entropy-based thresholding problem. Subsequently, we detected nuclei in contact with CD20+ (brown) pixels to identify the CD20+ cells. Finally, the total count of CD20+ B cells in the perivascular and peribronchiolar regions and the density of CD20+ B cells per square millimeter of peribronchiolar and perivascular segmented regions were calculated.

### Gene expression in lung tissue by microarray analysis

One lung lobe from Diversity Outbred mice (*n* = 117) was homogenized in TRIzol and stored at −80°C, and RNA was extracted using PureLink RNA Mini Kits (Life Technologies, Carlsbad, CA, USA). The Boston University Microarray and Sequencing Resource Core Facility (Boston, MA, USA) confirmed RNA quality and quantity and prepared and hybridized material to Mouse Gene (v.2.0) ST microarrays. Raw CEL files were normalized to produce gene-level expression values using the implementation of the robust multiarray average in the affy R package (v.1.62.0) and an Entrez Gene-specific probeset mapping (v.17.0.0) from the Molecular and Behavioral Neuroscience Institute (Brainarray) at the University of Michigan (31). All microarray data processing was performed using the R environment for statistical computing (v.3.6.0).

### Quantification of lung cytokines, chemokines, and anti-*M*. *tuberculosis* antibodies

After plating for *M. tuberculosis* colony-forming units, the remaining lung homogenates were aliquoted and stored at −80°C. Homogenates were thawed overnight at 4°C, serially diluted and assayed by sandwich enzyme-linked immunosorbent assay (ELISA) for cytokines and chemokines [CXCL5, CXCL2, CXCL1, tumor necrosis factor (TNF), matrix metalloproteinase 8 (MMP8), S100A8, interferon gamma (IFN-γ), IL12p40, interleukin (IL)-12p70, IL-10, and vascular endothelial growth factor (VEGF)] using antibody pairs and standards from R&D Systems (Minneapolis, MN, USA), Invitrogen (Carlsbad, CA, USA), eBioscience (San Diego, CA, USA), or BD Biosciences (San Jose, CA, USA), per kit instructions. A subset of these ELISA results was reported previously (22). The amount of immunoglobulin G (IgG) bound by *M. tuberculosis* cell wall fraction, *M. tuberculosis* culture filtrate proteins, *M. tuberculosis* antigen 85 complex, and *M. tuberculosis* ESAT-6:CFP-10 complex was quantified using in-house optimized ELISAs (22, 32). Briefly, high-binding immunoassay plates (Corning Costar #9018) were coated overnight in 100 µL/well of 1- to 5-µg/mL *M. tuberculosis* cell wall fraction (NR-14828); *M. tuberculosis* culture filtrate proteins (NR-14825); purified native *M. tuberculosis* antigen 85 complex (NR-14855); or recombinant purified *M. tuberculosis* ESAT-6 (NR-49424) and *M. tuberculosis* CFP-10 (NR-49425) obtained through BEI Resources, National Institute of Allergy and Infectious Diseases, National Institutes of Health. The following day, plates were blocked, and lung homogenates were serially diluted in 1% bovine serum albumin and incubated overnight at 4°C. After multiple washes, goat anti-mouse IgG-horseradish peroxidase (HRP) (Rockland Immunochemicals #610–13) was diluted per manufacturer’s instructions, incubated, washed multiple times, developed with TMB (Thermo Fisher or R&D Systems), stopped using 0.25-M HCl, and read at 450 nm using a BioTek Plate reader. The concentration of bound IgG was computed based on standard curves and four-parameter logistic regression models.

### Classification using cytokines, chemokines, and anti-*M*. *tuberculosis* IgG

#### Data

The linear classifier we used for the cytokines and chemokines, and IgG antibody-based classification can only be applied to tabular data sets that do not have missing entries. Therefore, we filtered for the mice with complete measurements for the 12 cytokines and antibodies: CXCL5, CXCL2, CXCL1, TNF, IFN-γ, IL-12, IL-10, MMP8, S100A8, VEGF, anti-*M*. *tuberculosis* cell wall (CW), and anti-*M*. *tuberculosis* CFP. This filtering yielded asymptomatic (*n* = 30), controllers (*n* = 48), and progressor mice (*n* = 38) from two independent experimental infections.

#### Classification methods

To discriminate between acute pulmonary TB in progressors, chronic pulmonary TB in controllers, and asymptomatic lung infection, we first used a linear classifier, L1 regularized logistic regression. The regularization term promotes sparse coefficients (33), and *λ* is selected through grid search among $\left\{0,10^{-2},10^{-1.95},\ldots ,10^{1.95},10^{2}\right\}.$ We used the scikit-learn implementation (34) of the logistic regression model. To mitigate the unbalanced classes, sample re-weighing with the “balanced” option of the scikit-learn library was used. We defined feature importance of a biomarker as the ratio of its (absolute) effect size (defined below) to the sum of all (absolute) effect sizes. More formally, let $\beta_{j}$ denote the effect size of the *j*th biomarker; its feature importance is given by $\frac{{|\beta }_{j}|}{\sum_{j=1}^{p}{|\beta }_{j}|}$, where *p* is the total number of biomarkers in that panel (i.e., 10 or 12). As an alternative method, we also tested a non-linear classifier, XGBoost (35). We used the python interface of the XGBoost and grid searched the two parameters: “learning_rate” ([1e-3, 1e-2]) and “n_estimators” ([3, 5, 100]). We fixed the parameter “max_depth” to 3 and used the default values for the remaining parameters. During training, we again used sample re-weighing. We reported the performance corresponding to the classifier’s best hyper-parameter using 30-fold cross-validation. Further details are discussed in the Supplemental Methods (36). The code used in the analysis will be made publicly available upon publication.

### Statistical analyses and other performance metrics

#### Data from *M. tuberculosis*-infected mice

Survival, weight loss, *M. tuberculosis* lung burden, total number of CD20+ cell, total number of CD20+ cell per square millimeter, and ELISA data were analyzed and graphed in GraphPad Prism (v.10.2.1 for CD20+ cell data, v.8.4.2 for all remaining data) with significance set at *P* < 0.05 and were adjusted for multiple comparisons. Survival curves were analyzed using log-rank (Mantel-Cox) test. To correct for optical density attributed to non-specificity in ELISAs measuring anti-*M*. *tuberculosis* CW, anti-*M*. *tuberculosis* CFP, and anti-*M*. *tuberculosis* Ag85, baseline correction was performed. First, outlier measurements in the non-infected group were detected using ROUT and excluded. Then the average of the cleaned data from non-infected group was calculated and subtracted from all measurements. Body weight data, lung *M. tuberculosis* burden, and lung cytokines, chemokines, and baseline-corrected antibody measurements were then tested for normal or log-normal distributions prior to one-way analysis of variance by Kruskal-Wallis with Dunn’s post-test or Brown-Forsythe and Welch’s with Dunnett’s T3 post-test as indicated in the figure legends.

#### Imaging biomarkers

Model performance was evaluated using overall sensitivity, specificity, and area under the receiver operating characteristics curve (AUC) of a 10-fold Monte-Carlo cross-validation. Ninety-five percent confidence intervals for each statistic were computed using bootstrapped samples of predictions (equal to the number of observations) with replacement (*n* = 1,000). Percentiles (97.5th and 2.5th) were taken as bounds for confidence intervals.

#### Microarray gene expression analyses

For each gene, AUC analysis was performed using R package pROC (v.1.18) (37). Corresponding *P* values to the AUC were calculated using one-sided Mann-Whitney *U*-statistic (38) with Python package statsmodels (v.0.13.2) (39). In the Supplemental Methods, we compared the statistically significant genes resulting from Mann-Whitney *U*-statistic with a parametric alternative, Welch’s *t*-test. For each classification problem, the directionality of the test was selected such that the gene expression values were statistically higher in the class with longer survival under the alternative hypothesis. Benjamini-Hochberg correction was applied separately to each classification problem to control the false discovery rate at 0.05 after filtering the genes without corresponding gene symbols. Clustermap is drawn using the python package seaborn (v.0.11.2) (40) with clustering metric “correlation.” Enrichr (https://amp.pharm.mssm.edu/Enrichr) (41) was used to identify Gene Ontology (GO) biological processes (v.2023) that were significantly overrepresented (adjusted *P* < 0.05) within an input set of official mouse gene symbols. A subset of microarray data and analyses from progressor mice were published elsewhere (22, 42), deposited in Gene Expression Omnibus (GEO) and assigned series ID GSE179417.

## RESULTS

### Survival, *M. tuberculosis* lung burden, and inflammatory biomarkers in infected mice

**Fig 1:**
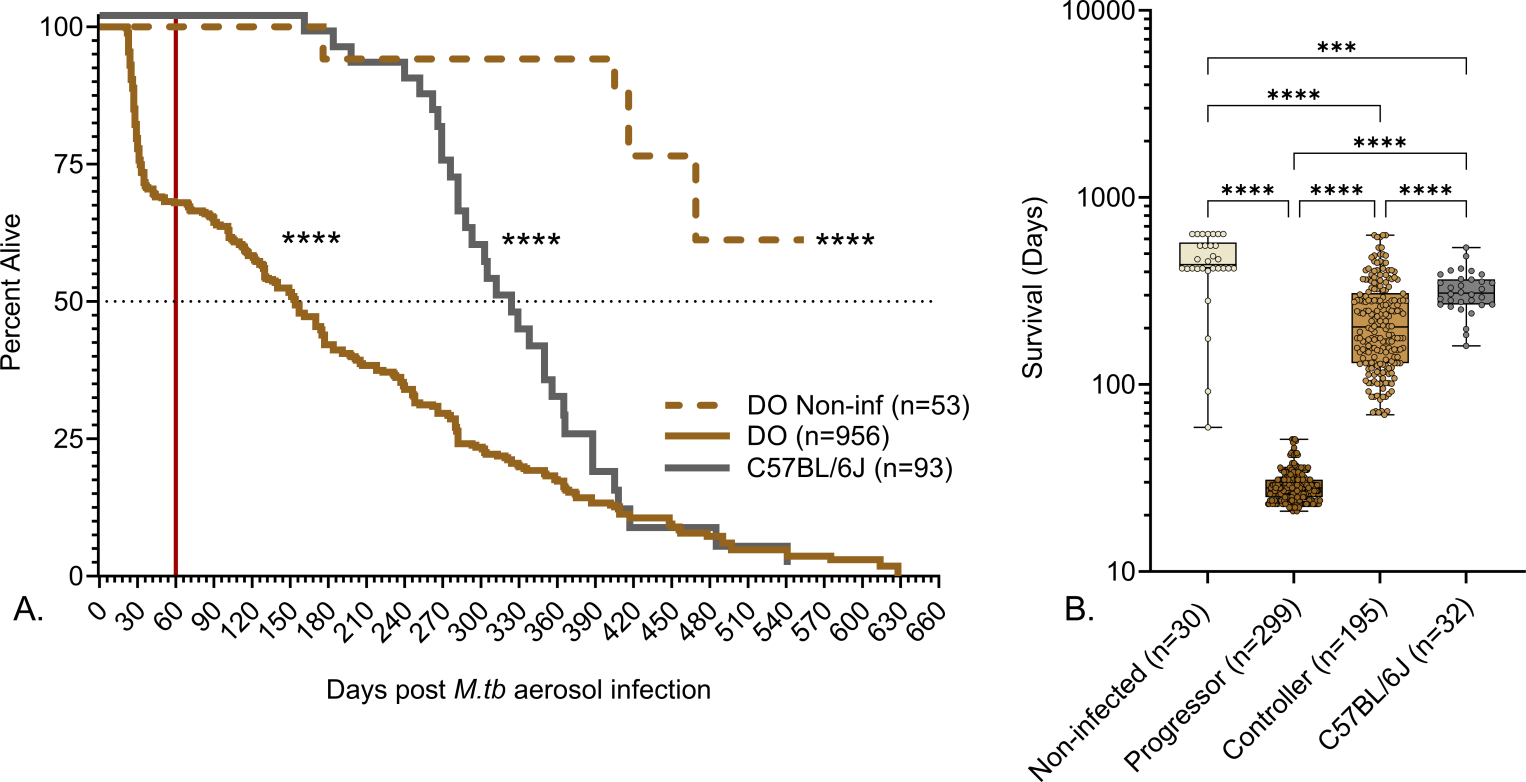
Survival of *M. tuberculosis*-infected mice. We infected 8- to 10-week-old, female Diversity Outbred (DO) mice and C57BL/6J mice with aerosolized *M. tuberculosis* bacilli and monitored as described in Materials and Methods. Non-infected, identically housed, and age- and gender-matched Diversity Outbred mice served as controls. Mice were euthanized at a predetermined timepoint, or if any one of three morbidity criteria developed a body condition score of <2, severe lethargy, or increased respiratory rate/effort. (A) The percent alive over time (cumulative survival) and the red vertical line mark 60 days post-infection when approximately 30% of Diversity Outbred mice succumbed to pulmonary TB due to early morbidity (progressors). Controllers survived at least 60 days without morbidity and succumbed later. Survival of non-infected Diversity Outbred mice (brown dashed line), infected Diversity Outbred (brown solid line), and infected inbred C57BL/6J (solid gray line) mice was significantly different by Mantel-Cox log-rank test. ****P < 0.0001. (B) A subset of 556 mice from panel A that were euthanized because of pulmonary TB-related morbidity (526 mice) or non-infected controls euthanized at the end of the experiment (30 mice). Groups are shown on the *X*-axis box-and-whiskers plots in panel B, with interquartile range with whiskers at the minimum and maximum. Statistical analysis was performed using Brown-Forsythe and Welch’s one-way analysis of variance followed by Dunnett’s T3 post-test. ****P < 0.0001.

A low dose of aerosolized *M. tuberculosis* (20 ± 12 bacilli) results in early morbidity and mortality in approximately one-third of Diversity Outbred mice that succumb to acute necrosuppurative pulmonary TB with high bacterial burden within 60 days. This phenotype is reproducible across sexes, institutions, aerosol infection methods, and strains of *M. tuberculosis* and is not due to immune deficiency (16, 18, 19, 22). *M. tuberculosis* infection significantly reduced survival of Diversity Outbred mice compared to identically housed, age- and sex-matched non-infected Diversity Outbred controls, and survival was significantly different from *M. tuberculosis-*infected C57BL/6J inbred mice, with approximately 25% of Diversity Outbred mice surviving longer than the median survival of C57BL/6J (Fig. 1A). The ~70% of Diversity Outbred mice that were more resistant to *M. tuberculosis* (i.e., controllers) survived longer than progressors (Fig. 1B). Table 1 summarizes forms of TB in humans (43–46) that may be comparable to relative susceptibility and resistance to *M. tuberculosis* in Diversity Outbred mice.

**TABLE 1:** Susceptibility to *M. tuberculosis* and forms of TB in humans and Diversity Outbred mice^*a*^

		Primary	Fulminant	Post-primary	Latent infection	Resisters
Human	Susceptibility	Variable	Highly susceptible	Susceptible	Resistant	Highly resistant
	Prevalence	Common	Rare	5%–10% of infected	90%–95% of infected	~20% of exposed
	Survival	Normal lifespan	Weeks	Years	Decades	Normal lifespan
	Symptoms	Usually none	Yes	Yes	No	No
	Radiography	Focal consolidation	Diffuse pneumonia	Focal, nodular, and cavitated	No lesions	No lesions
	Histology	Pneumonia and atelectasis	Fibrinous and necrotizing	Necrotic granulomas and cavities	Poorly defined	No lesions
	Antigen-specific immunoreactivity	Variable	Variable	Yes	Yes	No
	Cultivable bacilli	ND	Variable	Positive	BAL: neg. Lung tissue: ND	No

		Progressors (acute end-stage)	Controllers (chronic end-stage)	Asymptomatic
Diversity Outbred	Susceptibility	Highly susceptible	Susceptible	Model for lung resistance
	Prevalence	~30% of infected	~70% of infected	NA
	Survival	<60 days	>60 days	NA
	Symptoms	Yes	Yes	No
	Radiography	ND	ND	ND
	Histology	Fibrinosuppurative and necrotizing	Lymphohistiocytic with necrotic foci and rare cavities	Non-necrotizing and lymphocytic cuffs
	Antigen-specific immunoreactivity	Yes	Yes	Yes
	Cultivable bacilli	Yes	Yes	BAL: ND Lung tissue: pos.

^*a*^ The upper and lower halves of the table present the characteristics of TB in humans and Diversity Outbred mice, respectively. Columns correspond to different forms of TB and rows correspond to different properties. We present pairs of TB forms which share similar characteristics in the same column, i.e., fulminant and progressors (acute end stage), Post-primary and controllers (chronic end stage), latent infection, and asymptomatic. Such pairwise comparisons can have limitations (see Discussion) or not unavailable in general as the two forms, primary and resisters, do not have a corresponding class in the Diversity Outbred population. BAL, bronchoalveolar lavage; NA, not available; ND, not routinely done; neg., negative; pos., positive.

**Fig 2:**
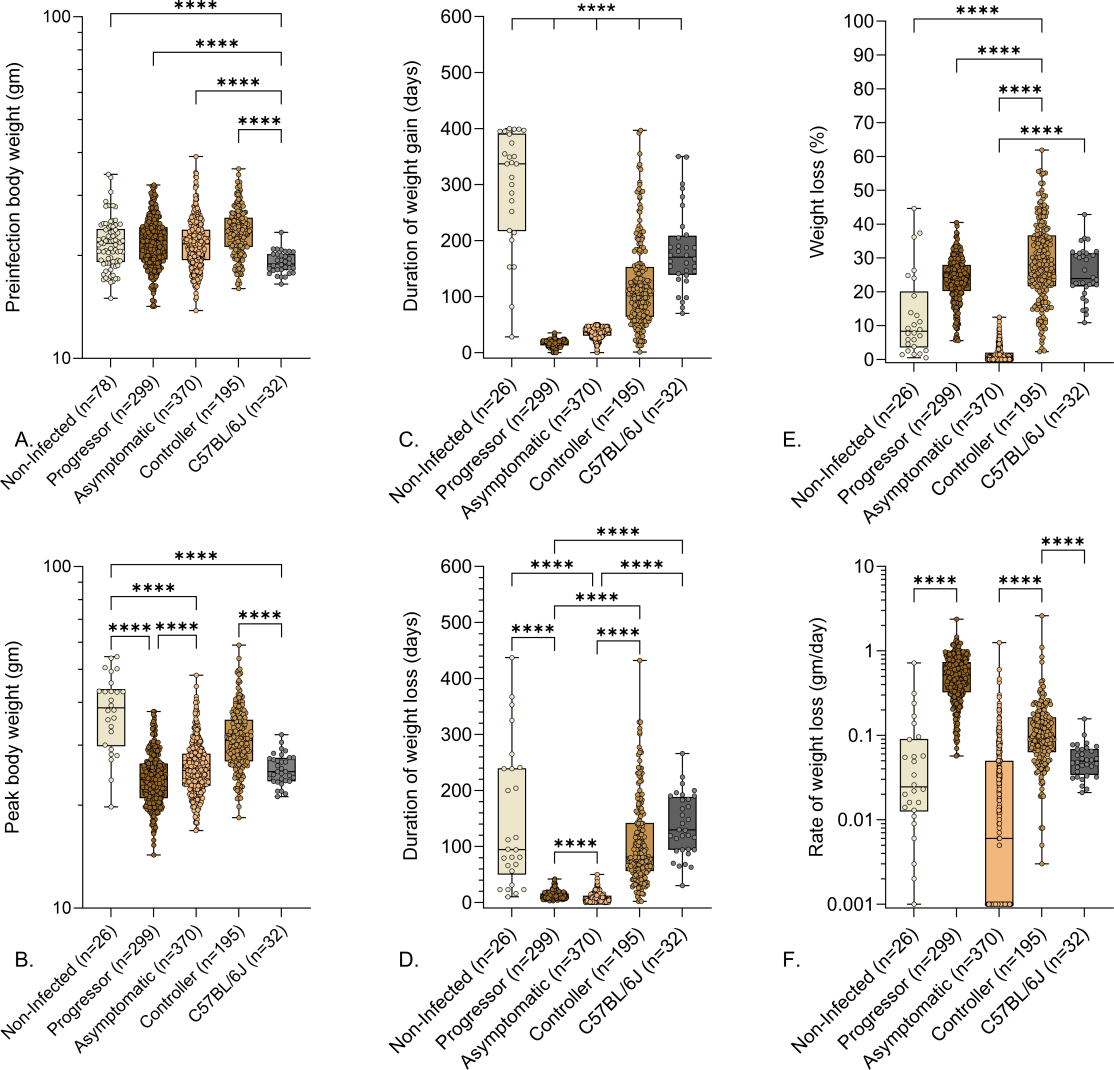
Preinfection body weights and weight-related indicators of pulmonary TB in *M. tuberculosis*-infected mice. We infected 8- to 10-week-old, female Diversity Outbred mice and C57BL/6J mice with aerosolized *M. tuberculosis* bacilli and monitored as described in Materials and Methods. Non-infected, identically housed, and age- and gender-matched Diversity Outbred mice served as controls. Mice were euthanized at a predetermined timepoint, or if any one of three morbidity criteria developed: body condition score of <2, severe lethargy, or increased respiratory rate/effort. (**A**) Preinfection body weights, (**B**) peak body weight achieved during infection, (**C**) duration of weight gain, (**D**) duration of weight loss, (**E**) percentage of peak body weight lost, and (**F**) rate of weight lost are shown. Box-and-whiskers plots in all panels show interquartile range with whiskers at the minimum and maximum. Group names and sample sizes are shown on the *X*-axis. Statistical analyses were performed using Brown-Forsythe and Welch’s one-way analysis of variance followed by Dunnett’s T3 post-test. Non-significant *P* values are not shown. ****P < 0.0001.

We speculated that resistance to *M. tuberculosis* could have been due to larger body size. However, retrospective analysis of preinfection body weight data failed to identify significant differences (Fig. 2A). As expected from inbreeding, age- and gender-matched C57BL/6J mice had a narrow preinfection weight range and, on average, weighed significantly less than Diversity Outbred mice (Fig. 2A). Controllers achieved higher body weights (Fig. 2B) due to normal growth while infected and longer duration of weight gain (Fig. 2C) prior to disease onset. Once disease onset occurred, controllers lost weight over a longer duration (Fig. 2D) and had a slower rate of weight loss or no weight loss (Fig. 2F). By euthanasia, both progressors and controllers had lost a similar percentage of weight (Fig. 2E), and controllers had a wider range of weight loss. Asymptomatic mice were euthanized before the end of their growth phase and thus achieved lower body weight than non-infected controls (Fig. 2B), and had a truncated duration of weight gain (Fig. 2C) and weight fluctuations without losing significant weight (Fig. 2D and E).

**Fig 3:**
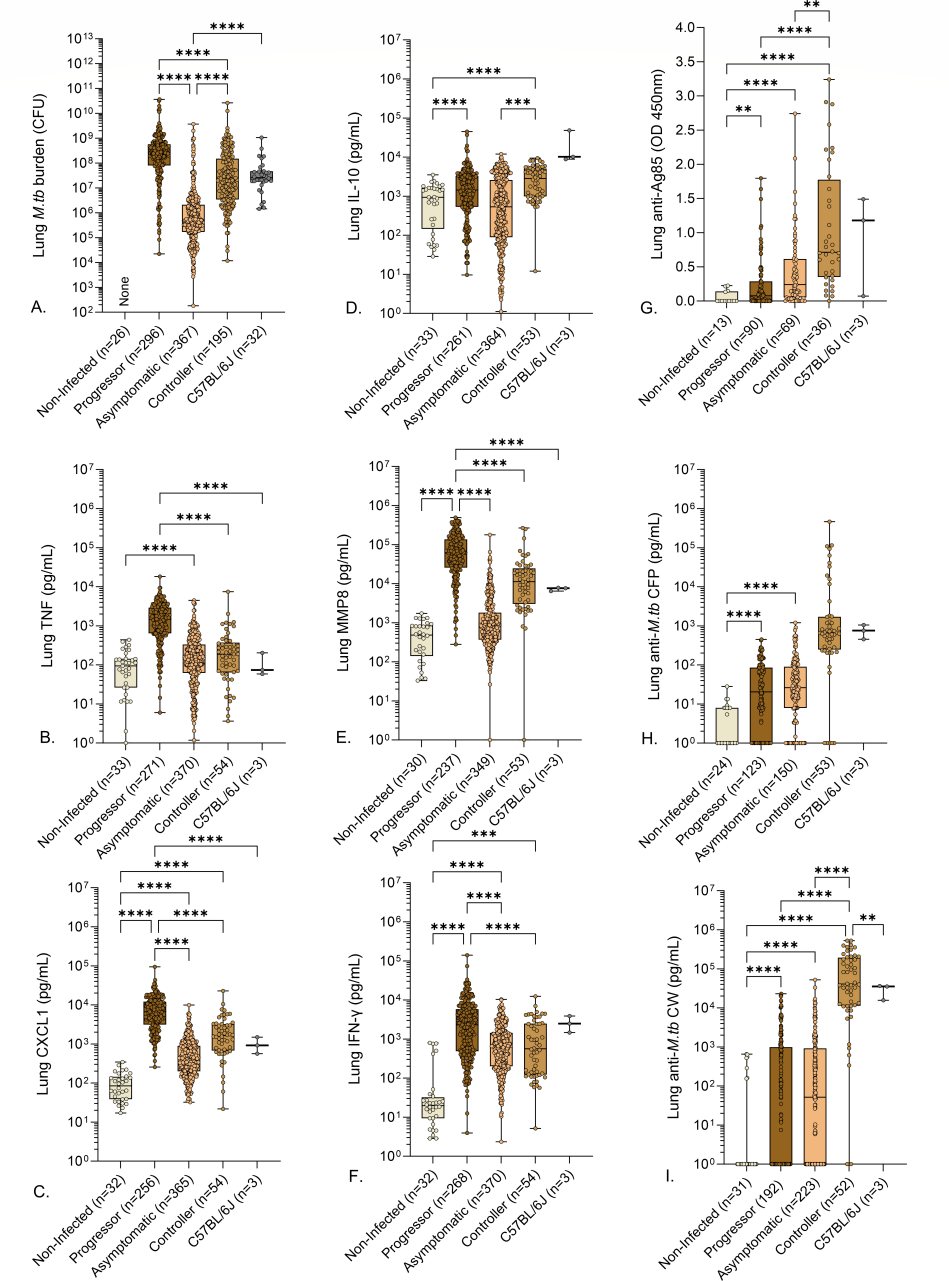
Lung *M. tuberculosis* burden, cytokines, chemokines, and anti-*M. tuberculosis* antibodies in *M. tuberculosis*-infected mice. We infected 8- to 10-week-old, female Diversity Outbred mice and C57BL/6J mice with aerosolized *M. tuberculosis* bacilli and monitored as described in Materials and Methods. Non-infected, identically housed, and age- and gender-matched Diversity Outbred mice served as controls. Mice were euthanized at a predetermined timepoint or if any one of three morbidity criteria developed: body condition score of <2, severe lethargy, or increased respiratory rate/effort. We quantified *M. tuberculosis* colony-forming units in the lungs (**A**) and measured eight lung proteins using sandwich ELISAs (**B–I**). Box-and-whiskers plots in all panels show interquartile range with whiskers at the minimum and maximum. Sample sizes are shown in the *X*-axis. Statistical analyses were performed using Kruskal-Wallis one-way analysis of variance (ANOVA) with Dunn’s multiple comparisons post-tests (A) or Brown-Forsythe and Welch’s one-way ANOVA followed by Dunnett’s T3 post-test (B–F). Non-significant *P* values are not shown. **P < 0.01, ***P < 0.001, ****P < 0.0001.

We expected controllers with end-stage pulmonary TB to achieve the same level of *M. tuberculosis* lung burden as progressors with end-stage pulmonary TB. Contrary to expectations, controllers had significantly lower *M. tuberculosis* burden than progressors (Fig. 3A). Likewise, we expected controllers and progressors to have similar levels of inflammatory mediators. However, except for IL-10, this also was not true. MMP8, CXCL1, TNF, and IFN-γ (22) were significantly lower in controllers compared to progressors (Fig. 3B, C, E and F) (Fig. 3D). These differences indicate that end-stage pulmonary TB in Diversity Outbred mice has two distinct pathogeneses: an “acute” form that is highly necrotizing, inflammatory, and promotes high *M. tuberculosis* bacillary growth, and a “chronic” form that is less inflammatory. Asymptomatic mice had significantly lower *M. tuberculosis* burden, CXCL1, and MMP8 and trend for lower levels of TNF, IL-10, and IFN-γ than progressors, and only lung *M. tuberculosis* burden and IL-10 were significantly lower between asymptomatic mice and controllers (Fig. 3A and D).

**Fig 4:**
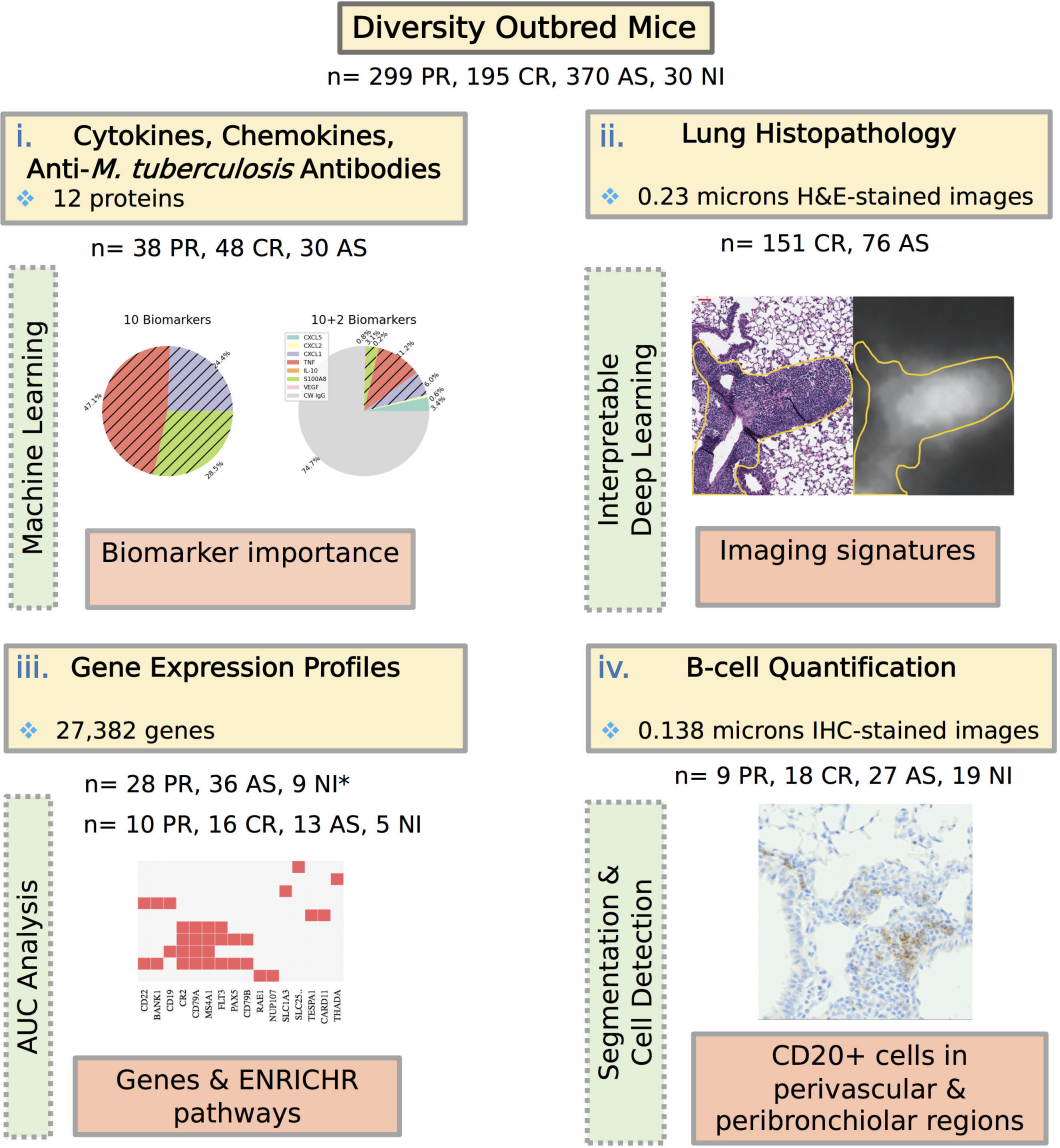
Four modalities, (i) protein biomarkers, (ii) H&E-stained lung tissue sections, (iii) gene expression profiles, and (iv) immunohistochemistry staining for B-cell quantification, are used to characterize asymptomatic *M. tuberculosis* lung infection in Diversity Outbred mice. We used statistical/machine learning approaches to quantify the feature importance of protein biomarkers; an interpretable deep-learning model to identify regions of H&E-stained slides, AUC analysis to filter genes for subsequent Enrichr pathway analysis, and entropy-based cell quantification for quantifying the CD20+ cells in segmented perivascular and peribronchiolar regions of immunohistochemistry (IHC)-stained images. *During the gene expression analysis, we used two separate data sets. The images corresponding to modalities ii and iv are included as examples to illustrate the analysis process and are presented with further details (see Fig. 7A and 9C, respectively). AS, asymptomatic; CR, controller; PR, progressor.

Next, we analyzed data from four different modalities: protein measurements, histopathology, gene expression profiles, and immunohistochemistry staining for B-cell quantification by machine learning and statistical methods to find signatures that could distinguish acute pulmonary TB (progressors) vs chronic pulmonary TB (controllers) vs asymptomatic infection (see Fig. 4). We hypothesized that unique features identified by models with high classification accuracy might provide insight into mechanisms of disease progression and of resistance to *M. tuberculosis.*

### A panel of lung cytokines, chemokines, and IgG antibodies classify acute TB and chronic pulmonary TB but not asymptomatic lung infection

**Fig 5:**
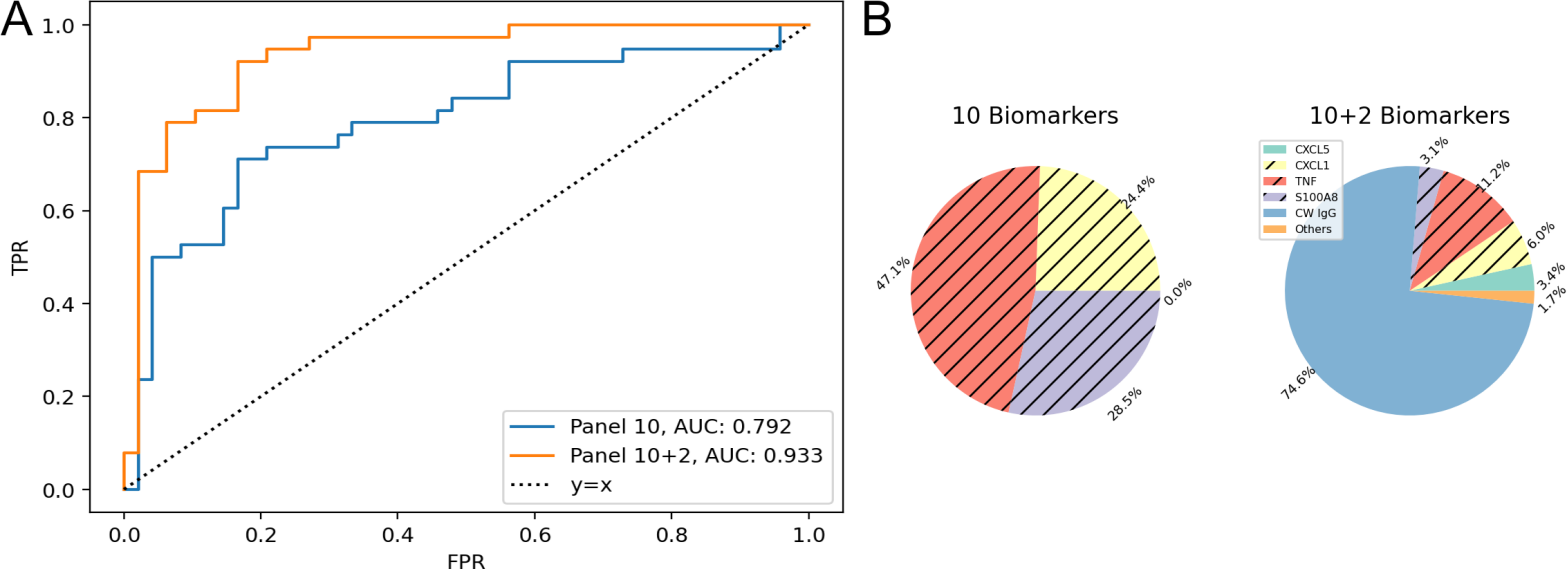
Feature importance analysis for classification between progressors and controllers using cytokine, chemokine, and anti-*M. tuberculosis* antibody measurements. (**A**) Receiver operating characteristic (ROC) curve comparison of the 10-biomarker panel (blue) and 12-biomarker panel (orange) for the progressor vs controller comparison. (**B**) Percent Importance of the different biomarkers in two panels. Logistic regression is the classifier and importance scores averaged over 30-fold. Biomarkers corresponding to the unhatched colors are associated with longer survival and vice versa for the hatched colors. Biomarkers with less than 1% importance are omitted. The feature scores for other comparisons are shown in Fig. S1 and S2.

To classify acute vs chronic end-stage pulmonary TB (i.e., progressors from controllers) vs asymptomatic infection, we analyzed a set of immune cytokines, chemokines, and growth factors by pairwise comparisons (Table 2). We first trained an L1-regularized logistic regression model using the following 10 cytokines, chemokines, and growth factors: CXCL5, CXCL2, CXCL1, TNF, IFN-γ, IL-12, IL-10, MMP8, S100A8, and VEGF. The classifier had high 30-fold cross-validation AUC (0.966) for progressor vs asymptomatic (Fig. S1) but performed relatively poorly (0.792 and 0.803) for comparisons against controllers (Table 2). When we included anti-*M. tuberculosis* CW IgG and anti-*M. tuberculosis* CFP IgG to the panel of lung proteins, the 30-fold cross-validation performance improved (Table 2). The improvement was highest for the progressor vs controller comparison in which the AUC increased from 0.792 to 0.933 (Fig. 5A). That improvement was attributed to anti-*M. tuberculosis* CW specifically, which had the highest average percentage of importance, while the other antibody, anti-*M. tuberculosis* CFP, was not used by the model (Fig. 5B). The classification between controllers and asymptomatic mice was the most challenging (AUC 0.83) (Table 2; Fig. S2). When an additional non-linear classifier, gradient tree boosting, was tested, the AUC did not improve (Table S1). All but one of the six panels performed with >90% accuracy when tested with (*n* = 22) non-infected Diversity Outbred mice previously unused during the training (Table S2). One panel, although successful for classifying between the two forms of end-stage pulmonary TB using antibodies, had low classification accuracy (32%) for the non-infected mice. That was because the non-infected Diversity Outbred mice had low levels of anti-*M. tuberculosis* CW (Fig. 3), which the classifier associates with lower survival (Fig. 5).

**TABLE 2:** Thirty-fold cross-validation performance of the two panels (10 and 10 + 2 antibodies) in three classification tasks (progressor vs controller, progressor vs asymptomatic, and controllers vs asymptomatic)^*a*^

Metric	PR vs CR		PR vs AS		CR vs AS	
	10	10 + 2	10	10 + 2	10	10 + 2
AUC	0.792	**0.933^*b*^**	0.966	**0.971**	0.803	**0.83**
Sensitivity (%)	57.9	**81.6**	**89.5**	86.8	68.8	68.8
Specificity (%)	**85.4**	83.3	**90**	**90**	76.7	**80**

^*a*^ Including antibodies improves the AUC in all three comparisons. The sensitivity is calculated with respect to Progressors in the first two comparisons and Controllers in the last comparison. Please refer to Fig. S3 for the confusion matrices. AS, asymptomatic; CR, controller; PR, progressor.

^*b*^ For each of the classification tasks and their corresponding metrics, the panel with the higher performance is highlighted in bold.

### Qualitative evaluation of lung granulomas yields insight into asymptomatic lung infection

**Fig 6:**
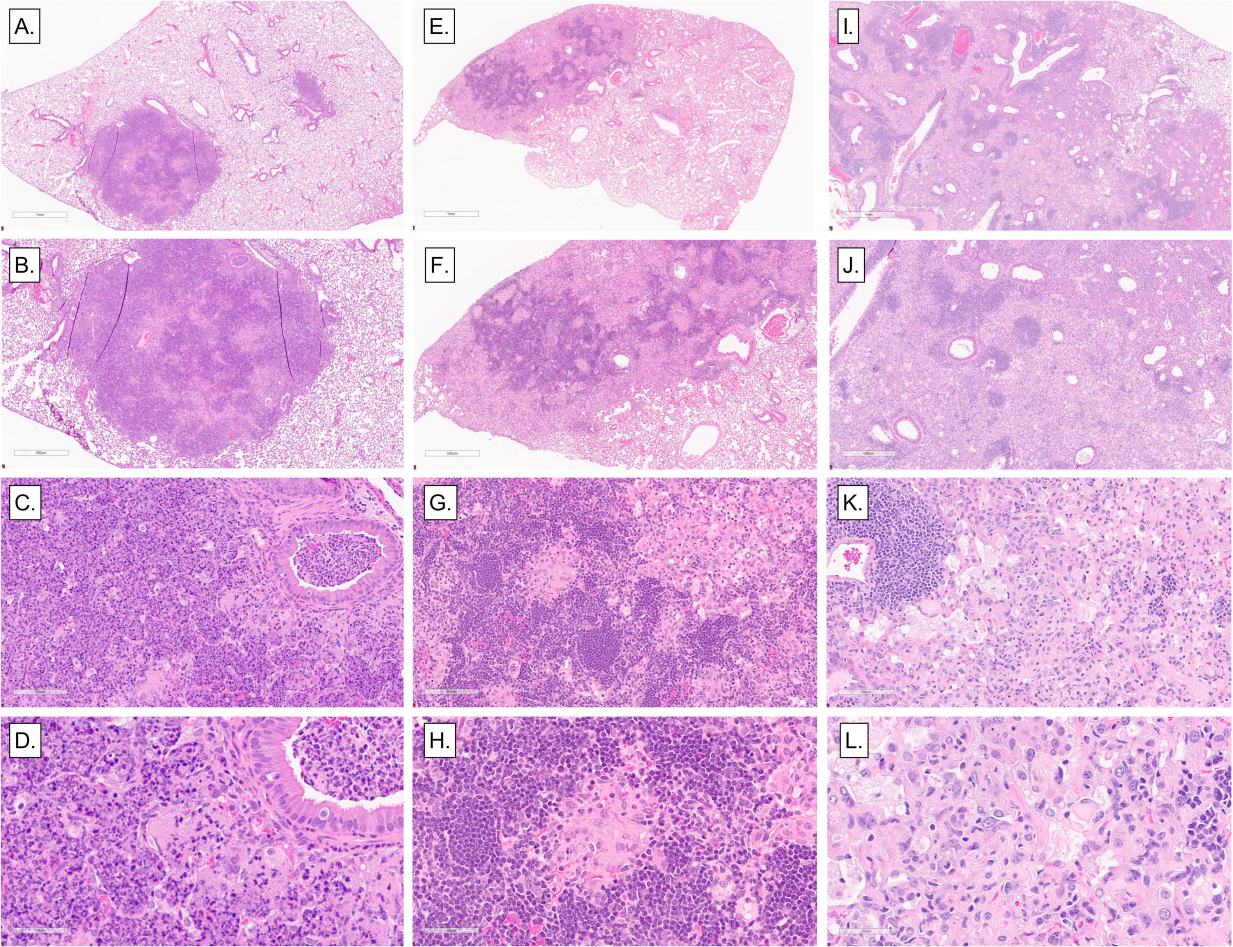
Representative histopathological lesions in the lungs of *M. tuberculosis-*infected Diversity Outbred mice. We infected 8- to 10-week-old, female Diversity Outbred mice with *M. tuberculosis* bacilli by inhalation. Lung lobes were fixed, stained, and sectioned for microscopic examination. (A–D) Representative necrosuppurative lung lesions with bronchiolar obstruction in progressors. (E–H) Non-necrotizing lymphohistiocytic lung lesions in asymptomatic mice. (I–L) Diffuse, non-necrotizing lesions with abundant macrophages, foamy macrophages, scattered lymphocytic foci, and a few cholesterol clefts in controllers. Magnifications are ×2, ×4, ×20, and ×40.

Anti-*M. tuberculosis* CW IgG improved the classification of progressors and controllers with acute and chronic end-stage pulmonary TB (Fig. 5) but not those with asymptomatic lung infection. To find features of asymptomatic lung infection, a board-certified veterinary pathologist (G.B.) examined lung sections of progressors, controllers, and mice with asymptomatic lung infection (Fig. 6). Qualitative differences in size, cellular infiltrates, and distribution of granulomas were noted, like previous publications (18, 22, 23, 42). The lungs of progressors contained coalescing fibrinous and necrosuppurative granulomas with abundant pyknotic nuclear debris in alveoli and obstructing bronchioles, and necrosis of alveolar septae (panels A through D), often with fibrin thrombosis of septal capillaries (not shown). In contrast, the lungs of asymptomatic mice typically contained small, discrete, non-necrotizing lesions with perivascular and peribronchiolar aggregates of lymphocytes and few neutrophils (panels E through H). The lungs of controllers typically contained diffuse, macrophage-dominated lesions, with many foamy macrophages, dense foci of lymphocytes and plasma cells, and occasional multinucleated giant cells (panels I through L), resembling end-stage pulmonary TB in the commonly used inbred mouse strain C57BL/6J (47). Additional features in controllers with end-stage pulmonary TB included cholesterol clefts, small pyogranulomas, septal fibrosis, cavitation with peripheral fibrosis, and bronchiolar obstruction with epithelial degeneration (not shown).

### A deep-learning neural network produced an accurate, human-interpretable imaging biomarker of asymptomatic lung infection

Qualitative histopathological evaluation of granulomas provided insight; however, quantification of unique histopathological granuloma features was not feasible. Therefore, we trained and validated a deep-learning neural network using multiple instance learning and attention-based pooling to (i) classify asymptomatic mice and controllers and (ii) identify regions within the granulomas where the model made classification decisions based on feature importance. Table 3 shows the model achieved close to 90% sensitivity and 70% specificity with AUC close to 90%, an improvement over the lung biomarker panel.

**TABLE 3:** Results of attention-based multiple instance learning model classification of asymptomatic mice and controllers

Metric	Cross-validation (*n* = 129)	Testing (*n* = 98)
AUC	0.987 (0.969–0.999)	0.884 (0.862–0.903)
Sensitivity	0.985 (0.949–1.000)	0.719 (0.632–0.804)
Specificity	0.906 (0.831–0.969)	0.899 (0.873–0.921)

**Fig 7:**
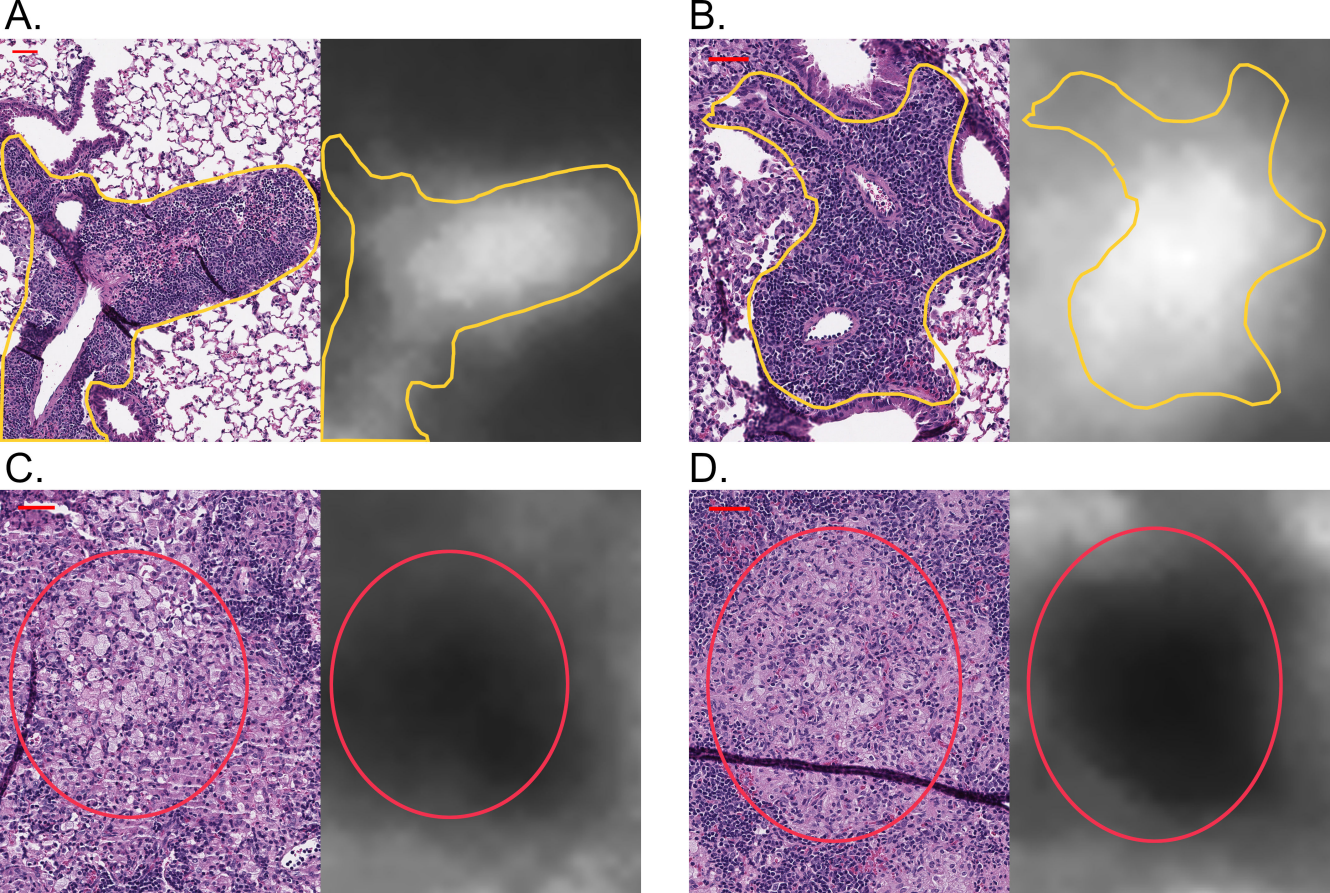
Perivascular and peribronchiolar lymphocytic cuffs are imaging biomarkers of asymptomatic lung infection and resistance to *M. tuberculosis*. For each panel pair, the H&E-stained lung tissue section is displayed on the left, and the corresponding attention weights for the same lung region are displayed on the right. (A and B) Representative examples where the deep-learning neural network found an imaging biomarker of asymptomatic lung infection in Diversity Outbred mice, and then a board-certified veterinary pathologist determined that the regions receiving the highest attention weights (white) corresponded to perivascular and peribronchiolar lymphoplasmacytic cuffs, outlined in yellow. In contrast, (C and D) representative examples within the granulomas where the regions received very low attention weights are in black, and then a board-certified veterinary pathologist determined that the regions receiving little attention were the macrophage-rich regions, encircled in red. A scale bar corresponding to 50 microns is displayed on each top left corner.

When we mapped attention weights back to the original images, the granuloma regions used as the basis for classifying asymptomatic lung infection was interpreted by a board-certified veterinary pathologist (G.B.) as perivascular and peribronchiolar lymphoplasmacytic cuffs (Fig. 7A and B; white areas, annotated in yellow). In contrast, the neural network did not weight granuloma regions that contained abundant macrophages, cholesterol clefts, or small pyogranulomas (Fig. 7C and D; black areas, annotated by red circles), which were characteristic of chronic pulmonary TB in controllers. Thus, the model identified an imaging biomarker (perivascular and peribronchiolar lymphoplasmacytic cuffs, a form of GrALT) is a diagnostically accurate granuloma feature of asymptomatic lung infection. We hypothesized this granuloma feature corresponded to unique functional responses capable of restricting *M. tuberculosis*.

### Gene expression analysis identifies functional correlates of asymptomatic infection in lung tissue

To identify functional correlates of asymptomatic lungs, we used transcriptional profiling and pathway analyses on two available data sets. One data set consisted of non-infected (*n* = 5), asymptomatic (*n* = 13), controller (*n* = 16), and progressor Diversity Outbred (*n* = 10) mice. The second data set consisted of non-infected (*n* = 9), asymptomatic (*n* = 36), and progressor Diversity Outbred (*n* = 28) mice.

Within each data set, we performed a one-sided AUC analysis (see Materials and Methods) comparing progressor and asymptomatic lung samples, which identified sets of 2,569 and 6,891 genes expressed at significantly (false discovery rate [FDR] *q* < 0.05) higher levels in the lungs of asymptomatic mice within data sets 1 and 2, respectively. These two sets contained 2,264 genes in common, with the average AUC values of the two data sets ranging from 0.743 to 0.969 with a median of 0.844 (File S1). This set of genes was input using Enrichr for pathway analysis, which identified 21 statistically significant (adjusted *P* < 0.05) Gene Ontology terms (Table S3).

In the same manner, we compared controller and progressor lungs using data set 1 and identified a set of 303 genes expressed at significantly (FDR *q* < 0.05) higher levels in the lungs of controllers. The AUC of the selected genes ranged from 0.888 to 1.0 with a median of 0.919 (File S1). Enrichr analysis identified four pathways containing statistically significant, highly expressed genes in controllers, and the pathways represent adaptive immunity, i.e., T-cell activation and B-cell receptor signaling pathway and antigen receptor-mediated signaling pathways (Table 4).

**TABLE 4:** Microarray Enrichr analysis resulting from the significantly different, highly expressed genes in the lungs of controllers with end-stage chronic pulmonary TB vs progressors with end-stage acute pulmonary TB^*a*^

Term	Overlap	Adjusted *P* value	Genes
Antigen receptor-mediated signaling pathway	14 of 134	<0.0001	IGHM, THEMIS, CD3E, LAX1, IGHG3, CD79B, CD79A, IGKC, CD8A, LCK, TRBC2, TRBC1, BLNK, and SKAP1
B-cell receptor signaling pathway	7 of 46	0.0032	CD79B, IGHG3, IGHM, and CD79A
			IGKC, LCK, and BLNK
T-cell activation	9 of 111	0.0189	CD2, PPP3CB, CD8A, IRF4, and LCK
			TRBC2, TRBC1, CD3E, and LY9
Regulation of lymphocyte activation	4 of 18	0.0387	KAT2A, LCK, SIT1, and IKZF3

^*a*^ Only the significant pathways (adjusted *P* < 0.05) are displayed.

None of the genes were significant at FDR *q* < 0.05 for asymptomatic vs controller lungs. Upon further inspection, however, we observed that genes with high diagnostic potential for the binary classification between asymptomatic and controller groups can include genes that are elevated in the non-infected lungs (Fig. S6), consistent with the histopathological finding that asymptomatically infected Diversity Outbred mice maintain a substantial fraction of normal lung tissue.

**Fig 8:**
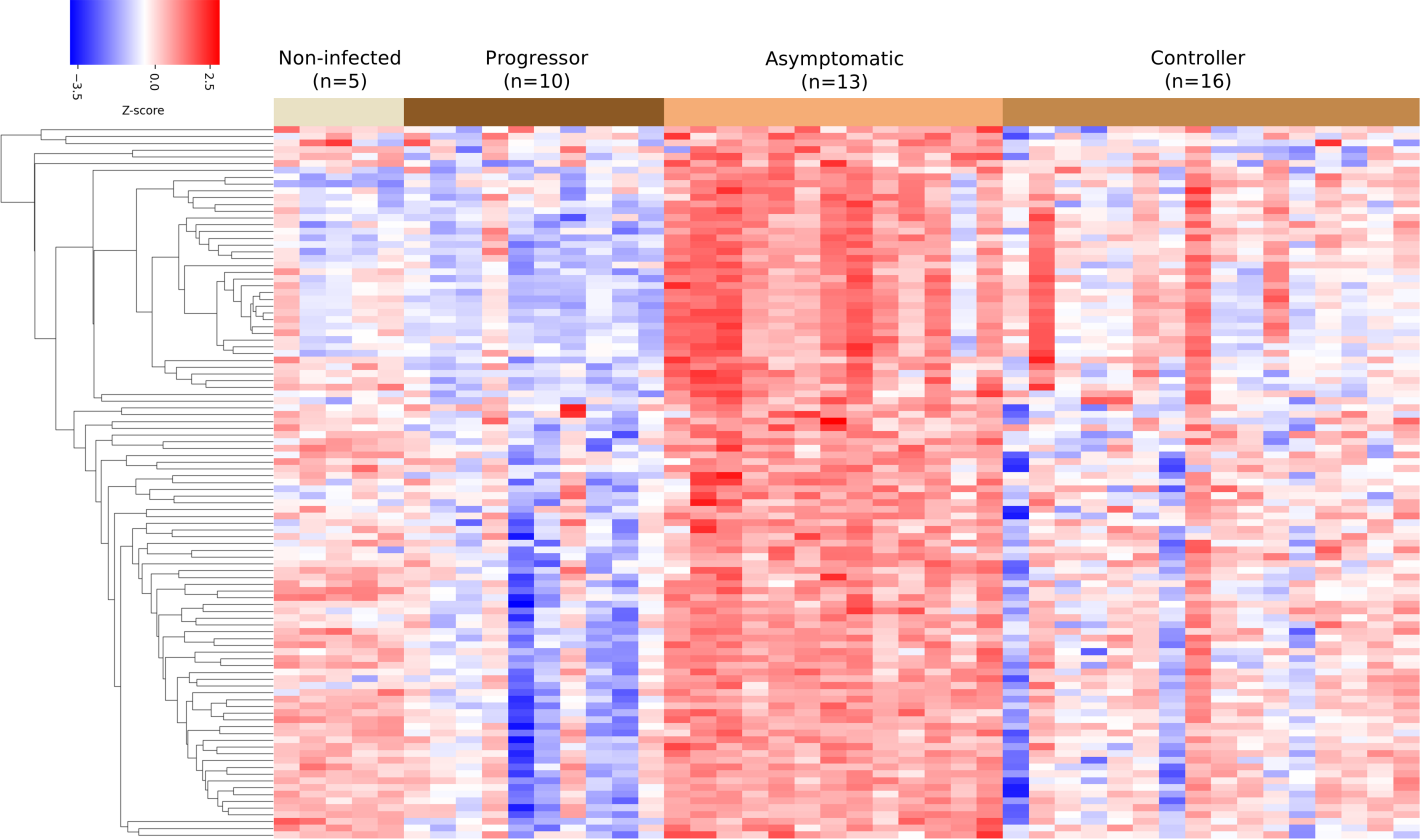
Cluster heat map of the 105 genes selected for the asymptomatic vs rest classification. Columns correspond to the (*n* = 44) mice used in the asymptomatic vs rest classification. Rows correspond to the identified 105 genes. Color indicates the *z*-score, which is calculated by first subtracting the row wise average from the gene expression value and next dividing it by the row wise standard deviation. Gene expression values are first log2 transformed.

To focus on finding unique transcriptional signatures, we compared lungs from asymptomatic mice to lungs from all other groups: controller, progressor, and non-infected using data set 1. This identified 105 genes that were expressed at significantly (FDR *q* < 0.05) higher levels in the lungs (Fig. 8). The AUC values of the identified genes ranged from 0.844 to 0.963 with a median of 0.864 ( File S1). Pathway analysis using Enrichr identified eight statistically significant (adjusted *P* < 0.05) GO terms associated with the 105 genes (Table 5). Five pathways are specific to B-cell functions. Two pathways indicate generic lymphocyte proliferation and differentiation and include B-cell genes (e.g., CD79A), and one pathway involves transcriptional responses. Overall, the unique pathways with highly expressed genes indicate that B-cell differentiation, proliferation, activation, and effector functions may be important for establishing asymptomatic lung infection and early resistance to *M. tuberculosis.*

**TABLE 5:** Microarray Enrichr analysis resulting from the genes selected for the asymptomatic lung classification vs other groups (progressor, controller, and non-infected)^*a*^

Term	Overlap	Adjusted *P* value	Genes
B-cell activation	8 of 92	<0.0001	CD79B, CD79A, CR2, FLT3, BANK1, PAX5, MS4A1, and CD22
B-cell differentiation	6 of 68	0.0004	CD79B, CD79A, CR2, FLT3, PAX5, and MS4A1
Lymphocyte differentiation	6 of 91	0.0014	CD79B, CD79A, CR2, FLT3, PAX5, and MS4A1
B-cell proliferation	4 of 31	0.0025	CD79A, CR2, CD19, and MS4A1
Lymphocyte proliferation	4 of 38	0.0046	CD79A, CR2, FLT3, and MS4A1
B-cell receptor signaling pathway	4 of 46	0.0082	CD79B, CD79A, CD19, and MS4A1
Regulation of B-cell activation	3 of 25	0.0211	BANK1, CD19, and CD22
Transcription-dependent tethering of RNA polymerase II gene DNA at nuclear periphery	2 of 6	0.0251	NUP107 and RAE1

^*a*^ Only the significant pathways (adjusted *P* < 0.05) are shown.

### Immunohistochemistry and quantitative image analysis shows more B cells in perivascular and peribronchiolar regions of asymptomatic lung infection

Gene expression profiles suggested that B cells were functionally important in asymptomatic control of *M. tuberculosis* lung infection but could not spatially locate B cells to the perivascular and peribronchiolar regions that were identified in the lung sections stained by carbol fuchsin and hematoxylin and eosin. To confirm, localize, and quantify B cells in the perivascular and peribronchiolar regions, we stained lung tissue sections from Diversity Outbred mice using immunohistochemistry to detect CD20. CD20 is specifically expressed by immature and mature B cells and is encoded by the gene *Ms4a1*, which was highly expressed (Table 5). Next, the peribronchiolar and perivascular regions were segmented, and areas were analyzed for CD20+ cells using entropy-based cell quantification combined with a deep learning-based nuclei detector, CellViT-SAM-H-x40.

**Fig 9:**
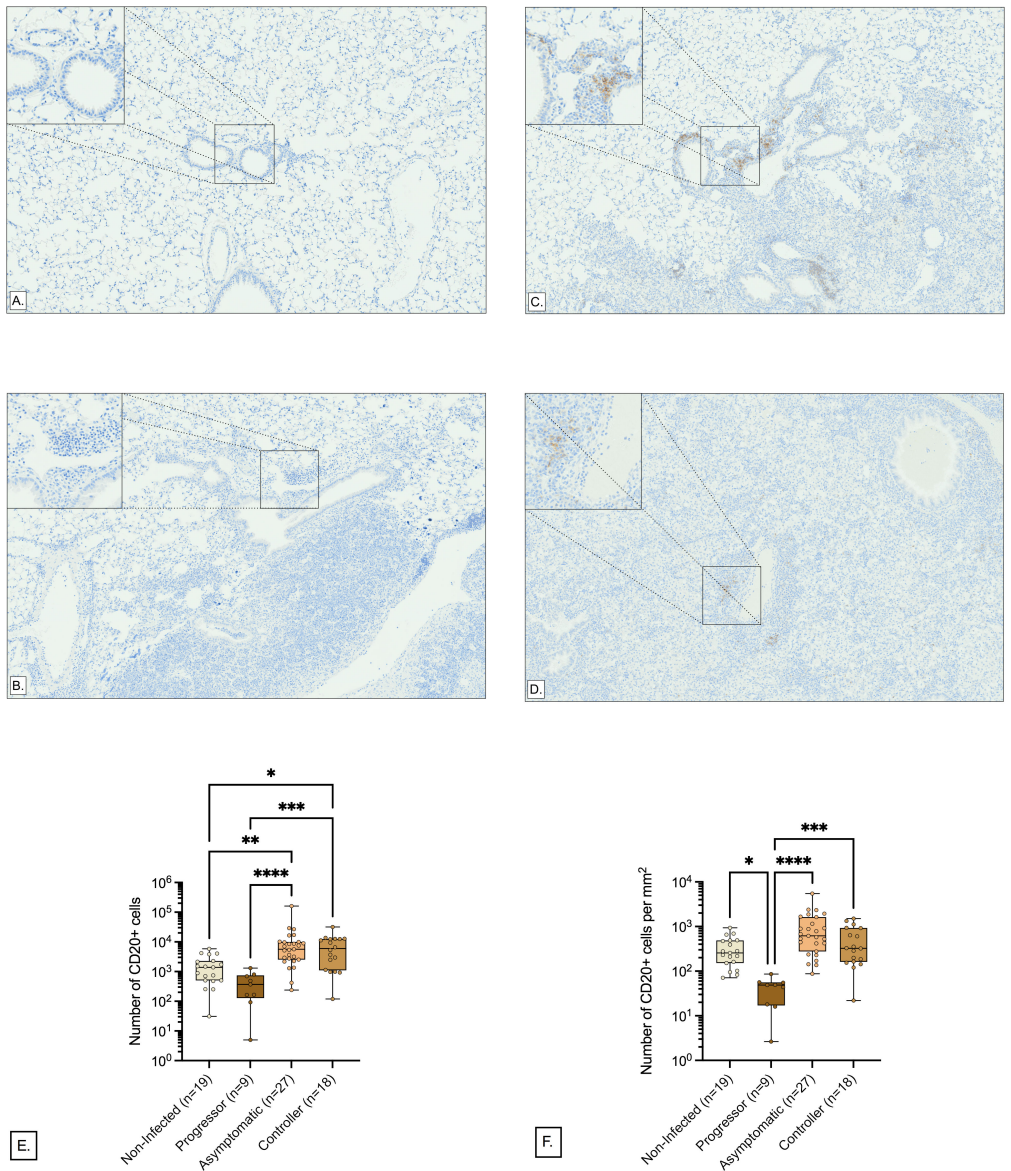
Distribution of CD20+ cells in the perivascular and peribronchiolar regions. To quantify the B cells within perivascular and peribronchiolar lymphocytic cuffs, we IHC-stained the lung tissue sections of *n* = 73 Diversity Outbred mice. The perivascular and peribronchiolar regions within the IHC images are segmented using Aiforia Create. In each segmented region, the CD20+ cells are detected by entropy-based cell quantification combined with a deep learning-based nuclei detector, CellViT-SAM-H-x40. (**A–D**) Representative perivascular and peribronchiolar regions of mice with CD20+ cell density close to their class medians. Each panel corresponds to a tissue section from a different susceptibility class: (**A**) non-infected, (B) progressor, (**C**) asymptomatic controller, and (D) controller. Magnification is ×80, and the high-magnification inserts are magnified ×400. The images were not altered in any way (i.e., not zoomed in or out) after extraction. (E and F) Visualization of the total number of CD20+ cells and density of CD20+ cells (cells per mm²) within the perivascular and peribronchiolar regions respectively. Box-and-whiskers plots in all panels show interquartile range with whiskers at the minimum and maximum. Each dot is one Diversity Outbred mouse and *n* = 27 Asymptomatic, *n* = 9 Progressor, *n* = 18 Controller, and *n* = 19 non-infected mice are shown. Statistical analyses were performed using Kruskal-Wallis one-way ANOVA with Dunn’s multiple comparisons post-tests (E and F). Non-significant *P* values are not shown. **P < 0.01, ***P < 0.001, ****P < 0.0001, * *P*<0.05.

Pathologist evaluation confirmed positive and negative assay controls worked as expected, and confirmed the presence of perivascular and peribronchiolar CD20+ cells in all lung tissue sections. Representative images from non-infected, progressor, asymptomatic, and controller lungs at low and high magnification show the brown staining for CD20 is most evident around bronchioles and blood vessels of asymptomatic and controller mice (Fig. 9A through D). The total number of CD20+ cells in the peribronchiolar and perivascular regions showed statistically significant differences between the asymptomatic (*n* = 27), progressor (*n* = 9), controller (*n* = 18), and non-infected Diversity Outbred mice (*n* = 19) (Fig. 9E). Controllers and asymptomatic mice had higher total CD20+ cell counts, and both groups were statistically different compared to the progressor and the non-infected groups. The density of CD20+ cells per square millimeter of peribronchiolar and perivascular regions also varied significantly between the four groups (Fig. 9F), with progressor lungs having significantly lower CD20+ cell density than all other groups and a trend for highest CD20+ cell density in asymptomatic that was not statistically significant. The median value of the density of CD20+ cells was the lowest in progressors, 48.66 cells/mm^2^, followed by non-infected mice with 256.0 cells/mm^2^, controllers with 324.9 cells/mm^2^, and asymptomatic mice with the highest median value, 619.2 cells/mm^2^. Overall, these quantitative immunohistochemistry results validate the gene expression profiles of asymptomatic lung infection and spatially locate B cells to the discriminatory imaging biomarker (perivascular and peribronchiolar lymphocytic cuffs) of asymptomatic lung infection.

## DISCUSSION

Although pulmonary TB remains a global health problem with high morbidity and mortality, most humans develop asymptomatic infection of various forms (e.g., primary TB, latent TB infection, and resisters). Only a minority of infected humans develop any of the symptomatic disease forms (e.g., progressive primary TB, miliary TB, fulminant TB, or active pulmonary TB) (1, 6, 48–51). Because infection is often silent and lung tissues from naturally resistant humans are not readily available, mechanisms of lung resistance are difficult to model and identify. A growing body of evidence supports the use of the Diversity Outbred mouse population to model disease states of pulmonary TB in biomarker discovery, gene expression signatures, and pathogenesis (14–19, 22, 32, 52). However, fewer studies focus on resistance to *M. tuberculosis*, which occurs in asymptomatic infection. The novelty of this work is finding unique features of lung resistance to *M. tuberculosis* by using the Diversity Outbred mouse population and multimodal computational approaches.

To discover signatures of resistance to *M. tuberculosis* infection, we performed long-term survival studies in the Diversity Outbred mouse population and showed survival was bimodal: less than 60 days or greater than 60 days. Interestingly, no infected mice reached the median survival of non-infected mice, and even the most resistant Diversity Outbred mouse eventually succumbed to chronic progressive pulmonary TB, as occurs in commonly used inbred strains of mice (47). We determined that the cytokines and chemokines that accurately classified progressors with acute pulmonary TB from non-progressors (22) could not distinguish progressors from controllers with chronic pulmonary TB but that the addition of anti-*M. tuberculosis* CW IgG significantly improved diagnostic accuracy. None of cytokines, chemokines, growth factors, or antibodies in our data set produced a biomarker panel that could accurately classify asymptomatic lung infection. This interesting result is consistent with historical challenges to accurately diagnose latent TB infection in humans by using skin tests, interferon gamma responses, and antibody-based serological tests (53–59). However, recent studies are more promising as specific types of antibodies have more diagnostic power (60–62).

In our studies, histopathological analyses combined with gene expression were much more useful to find key features of asymptomatic lung infection that have diagnostic value and provide mechanistic insight. Our deep-learning model automatically identified a granuloma feature specific to asymptomatic *M. tuberculosis* lung infection, which was interpreted by a pathologist as perivascular and peribronchiolar lymphocytic cuffs. This histopathological granuloma feature aligns with a large body of prior work in inbred mice, non-human primates, and natural experiments in humans (e.g., humans with acquired immune deficiency, or with genetic immune deficiencies), indicating that CD4 T lymphocytes and their effector molecules are required for resistance to *M. tuberculosis* (63). We therefore expected that lung tissue from asymptomatically infected Diversity Outbred mice would contain highly expressed genes and gene expression pathways indicative of CD4 T cell functions. However, we were surprised to find that the differentially expressed genes in lungs of Diversity Outbred mice with asymptomatic *M. tuberculosis* infection corresponded to B-cell functions and signaling, not to CD4 T-cell functions and signaling. Of the eight upregulated pathways in lungs of asymptomatically infected Diversity Outbred mice, seven involved B-cell differentiation, proliferation, activation, or effector functions. None of the significant pathways were specific for T lymphocytes or CD4 T cells of any subtype.

We compared our gene expression results with a 2020 study by Ahmed et al*.* (19), which used RNAseq to identify differences between Diversity Outbred mice with a high-risk disease score (*n* = 16), low-risk disease score (*n* = 13), and non-infected (*n* = 10). Of the 105 genes we identified in the lungs of asymptomatically infected Diversity Outbred mice, 14 were also reported by Ahmed et al. as significantly overexpressed in low-risk vs high-risk disease scores, and 52 were significantly overexpressed in the low-risk disease score vs non-infected (19). The different results may reflect differences in methods (microarray vs RNAseq) and sample size (hundreds vs tens). However, more importantly, among the matching sets of 14 and 52 highly expressed genes, 10 overlapped: *Pax5*, *Zfp318*, *Thada*, *Ralgps2*, *Dclk2*, *Itpr2*, *Cyb561a3*, *Dock8*, *F8*, and *Bach2.* Many of these genes transcriptionally regulate B-cell differentiation and immunoglobulin production. Esaulova et al. reported similar findings in a 2021 study that B-cell follicles were smaller in the lungs of macaques with pulmonary TB (*n* = 5) compared to those with latent TB infection (*n* = 2), and a negative correlation between the B-cell follicle size and lung *M. tuberculosis* burden (64) supported a protective role of inducible bronchiolar-associated lymphoid tissue. Their single-cell RNAseq analysis indicated the relative number of CD79A+ B cells was higher in the pulmonary TB compared with latent TB infection (64). Our results suggest the opposite: lungs of asymptomatically infected Diversity mice had significantly higher expression of *Cd79a* as well as other B-cell genes, including *Ms4a1* that encodes CD20. We also observed more CD20+ B cells in perivascular and peribronchiolar regions.

Our studies examining resistance to *M. tuberculosis* using protein biomarkers, lung histopathology, deep-learning neural networks, gene expression profiles, and immunohistochemistry provide insight, but there are limitations. One limitation is that Diversity Outbred mouse population does not model all forms of TB in humans, likely because *M. tuberculosis* is a human-adapted bacillus and because humans and mice have different-sized lungs, leading to clinical disease at different-sized lesions. For example, the lungs of an asymptomatic human could readily tolerate a 1-cm^3^ granuloma, but that same-sized lesion would cause mortality in a mouse. Although we have nearly 900 mice available for survival and body weights, we have gaps in the data sets, and subsequent analyses used smaller subsets, often 100–200 samples. In part, these data gaps reflect smaller volumes, for example, in biomarker panel studies. The gene expression profiles had 117 samples available, and second sets of gene expression profiles remain in processing and will become available for future studies. Lastly, this work did not explore joint analysis of different modalities because using either gene expression profiles or histopathology slides alone enabled accurate classification between acute pulmonary TB in progressors, chronic pulmonary TB in controllers, and the lungs from asymptomatically infected mice. A future direction can be capturing the intermodal relationships for identifying more complex resistance signatures and boosting the diagnostic accuracy.

Overall, our results show two main findings that have important implications. First, there are two distinct forms of end-stage pulmonary TB in Diversity Outbred mice, which can inform the pathogenesis of pulmonary TB in humans and support research to discover and host-directed therapies against these two forms of TB. Second, by applying novel computational approaches, image analysis, and lung transcriptional profiles, we found granuloma regions of perivascular and peribronchiolar lymphocytic cuffs specific to asymptomatic lung infection and lung functional responses, which show B cells may be important to establish asymptomatic *M. tuberculosis* lung infection in genetically heterogenous populations. Future studies using the Diversity Outbred mouse population can define the genetic control upstream of B-cell responses to *M. tuberculosis* to improve our understanding of how genotype controlled responses restrict *M. tuberculosis*.

## Data Availability

The data discussed in this publication have been deposited in NCBI's Gene Expression Omnibus and are accessible through GEO Series accession number GSE266564.

## SUPPLEMENTAL MATERIAL

File S1 iai.00263-23-s0001.xlsx Additional details regarding the gene expression analysis.

Supplemental material iai.00263-23-s0002.docx Figures S1 to S6, Tables S1 to S3, and supplemental methods.

ASM does not own the copyrights to Supplemental Material that may be linked to, or accessed through, an article. The authors have granted ASM a non-exclusive, world-wide license to publish the Supplemental Material files. Please contact the corresponding author directly for reuse.

## References

[1] Organization WH. 2021. Global tuberculosis report 2021. World Health Organization, Geneva, Switzerland.

[2] Hunter RL, Actor JK, Hwang SA, Karev V, Jagannath C. 2014. Pathogenesis of post primary tuberculosis: immunity and hypersensitivity in the development of cavities. Ann Clin Lab Sci 44:365–387. 25361920

[3] Adzic-Vukicevic T, Barac A, Ilic AD, Jankovic R, Hadzi-Djokic J, Pesut D. 2017. First reported case of fulminant TB with progression of infection from lungs to the genitourinary region. Rev Inst Med Trop Sao Paulo 59:e20. doi:10.1590/S1678-9946201759020 28423095 PMC5440999

[4] Peck-Radosavljevic M, Prokesch R, Schmid K, Frossard M, Wichlas M, Gendo A, Gangl A, Madl C. 1999. Fulminant lethal tuberculous pneumonia (Sepsis tuberculosis gravissima) with ARDS in a non-immunocompromised western European middle-aged man. Wien Klin Wochenschr 111:157–160. 10192149

[5] Dickens P. 1991. Fulminant tuberculous bronchopneumonia in a young Hong Kong Chinese woman. Pathology 23:248–249. doi:10.3109/00313029109063576 1780191

[6] Verrall AJ, Netea MG, Alisjahbana B, Hill PC, van Crevel R. 2014. Early clearance of Mycobacterium tuberculosis: a new frontier in prevention. Immunology 141:506–513. doi:10.1111/imm.12223 24754048 PMC3956425

[7] Mack U, Migliori GB, Sester M, Rieder HL, Ehlers S, Goletti D, Bossink A, Magdorf K, Hölscher C, Kampmann B, Arend SM, Detjen A, Bothamley G, Zellweger JP, Milburn H, Diel R, Ravn P, Cobelens F, Cardona PJ, Kan B, Solovic I, Duarte R, Cirillo DM, C. Lange, TBNET. 2009. LTBI: latent tuberculosis infection or lasting immune responses to M. tuberculosis? A TBNET consensus statement. Eur Respir J 33:956–973. doi:10.1183/09031936.00120908 19407047

[8] Wang Q, Guo S, Wei X, Dong Q, Xu N, Li H, Zhao J, Sun Q. 2022. Global prevalence, treatment and outcome of tuberculosis and COVID-19 coinfection: a systematic review and meta-analysis (from November 2019 to March 2021). BMJ Open 12:e059396. doi:10.1136/bmjopen-2021-059396 PMC9213780 35725250

[9] Aibana O, Huang C-C, Aboud S, Arnedo-Pena A, Becerra MC, Bellido-Blasco JB, Bhosale R, Calderon R, Chiang S, Contreras C, et al.. 2019. Vitamin D status and risk of incident tuberculosis disease: a nested case-control study, systematic review, and individual-participant data meta-analysis. PLoS Med 16:e1002907. doi:10.1371/journal.pmed.1002907 31509529 PMC6738590

[10] Silva DR, Muñoz-Torrico M, Duarte R, Galvão T, Bonini EH, Arbex FF, Arbex MA, Augusto VM, Rabahi MF, Mello F de Q. 2018. Risk factors for tuberculosis: diabetes, smoking, alcohol use, and the use of other drugs. J Bras Pneumol 44:145–152. doi:10.1590/s1806-37562017000000443 29791552 PMC6044656

[11] Patra J, Jha P, Rehm J, Suraweera W. 2014. Tobacco smoking, alcohol drinking, diabetes, low body mass index and the risk of self-reported symptoms of active tuberculosis: individual participant data (IPD) meta-analyses of 72,684 individuals in 14 high tuberculosis burden countries. PLoS One 9:e96433. doi:10.1371/journal.pone.0096433 24789311 PMC4008623

[12] Abel L, El-Baghdadi J, Bousfiha AA, Casanova JL, Schurr E. 2014. Human genetics of tuberculosis: a long and winding road. Philos Trans R Soc Lond B Biol Sci 369:20130428. doi:10.1098/rstb.2013.0428 24821915 PMC4024222

[13] Naranbhai V. 2016. The role of host genetics (and genomics) in tuberculosis. Microbiol Spectr 4. doi:10.1128/microbiolspec.TBTB2-0011-2016 27787193

[14] Smith CM, Baker RE, Proulx MK, Mishra BB, Long JE, Park SW, Lee H-N, Kiritsy MC, Bellerose MM, Olive AJ, et al.. 2022. Host-pathogen genetic interactions underlie tuberculosis susceptibility in genetically diverse mice. Elife 11:e74419. doi:10.7554/eLife.74419 35112666 PMC8846590

[15] Smith CM, Proulx MK, Olive AJ, Laddy D, Mishra BB, Moss C, Gutierrez NM, Bellerose MM, Barreira-Silva P, Phuah JY, Baker RE, Behar SM, Kornfeld H, Evans TG, Beamer G, Sassetti CM. 2016. Tuberculosis susceptibility and vaccine protection are independently controlled by host genotype. mBio 7:e01516-16. doi:10.1128/mBio.01516-16 PMC5030360 27651361

[16] Kurtz SL, Rossi AP, Beamer GL, Gatti DM, Kramnik I, Elkins KL. 2020. The Diversity Outbred mouse population is an improved animal model of vaccination against tuberculosis that reflects heterogeneity of protection. mSphere 5:e00097-20. doi:10.1128/mSphere.00097-20 32295871 PMC7160682

[17] Gopal R, Monin L, Torres D, Slight S, Mehra S, McKenna KC, Fallert Junecko BA, Reinhart TA, Kolls J, Báez-Saldaña R, et al.. 2013. S100A8/A9 proteins mediate neutrophilic inflammation and lung pathology during tuberculosis. Am J Respir Crit Care Med 188:1137–1146. doi:10.1164/rccm.201304-0803OC 24047412 PMC3863739

[18] Niazi MKK, Dhulekar N, Schmidt D, Major S, Cooper R, Abeijon C, Gatti DM, Kramnik I, Yener B, Gurcan M, Beamer G. 2015. Lung necrosis and neutrophils reflect common pathways of susceptibility to Mycobacterium tuberculosis in genetically diverse, immune-competent mice. Dis Model Mech 8:1141–1153. doi:10.1242/dmm.020867 26204894 PMC4582107

[19] Ahmed M, Thirunavukkarasu S, Rosa BA, Thomas KA, Das S, Rangel-Moreno J, Lu L, Mehra S, Mbandi SK, Thackray LB, Diamond MS, Murphy KM, Means T, Martin J, Kaushal D, Scriba TJ, Mitreva M, Khader SA. 2020. Immune correlates of tuberculosis disease and risk translate across species. Sci Transl Med 12:eaay0233. doi:10.1126/scitranslmed.aay0233 31996462 PMC7354419

[20] Kus P, Gurcan MN, Beamer G. 2019. Automatic detection of granuloma necrosis in pulmonary tuberculosis using a two-phase algorithm: 2D-TB. Microorganisms 7:661. doi:10.3390/microorganisms7120661 31817882 PMC6956251

[21] Niazi MKK, Beamer G, Gurcan MN. 2017. A computational framework to detect normal and tuberculosis infected lung from H and E-stained whole slide images. Proc SPIE Int Soc Opt Eng 10140:101400J. doi:10.1117/12.2255627 PMC10860644 38347946

[22] Koyuncu D, Niazi MKK, Tavolara T, Abeijon C, Ginese ML, Liao Y, Mark C, Specht A, Gower AC, Restrepo BI, Gatti DM, Kramnik I, Gurcan M, Yener B, Beamer G. 2021. CXCL1: a new diagnostic biomarker for human tuberculosis discovered using Diversity Outbred mice. PLoS Pathog 17:e1009773. doi:10.1371/journal.ppat.1009773 34403447 PMC8423361

[23] Tavolara TE, Niazi MKK, Ginese M, Piedra-Mora C, Gatti DM, Beamer G, Gurcan MN. 2020. Automatic discovery of clinically interpretable imaging biomarkers for Mycobacterium tuberculosis supersusceptibility using deep learning. EBioMedicine 62:103094. doi:10.1016/j.ebiom.2020.103094 33166789 PMC7658666

[24] Harrison DE, Astle CM, Niazi MKK, Major S, Beamer GL. 2014. Genetically diverse mice are novel and valuable models of age-associated susceptibility to Mycobacterium tuberculosis. Immun Ageing 11:24. doi:10.1186/s12979-014-0024-6 25606048 PMC4299371

[25] Ullman-Culleré MH, Foltz CJ. 1999. Body condition scoring: a rapid and accurate method for assessing health status in mice. Lab Anim Sci 49:319–323. 10403450

[26] Ilse M, Tomczak J, Welling M. Attention-based deep multiple instance learning PMLR, p 2127–2136

[27] Xu Q-S, Liang Y-Z. 2001. Monte Carlo cross validation. Chemom Intell Lab Syst 56:1–11. doi:10.1016/S0169-7439(00)00122-2

[28] Hörst F, Rempe M, Heine L, Seibold C, Keyl J, Baldini G, Ugurel S, Siveke J, Grünwald B, Egger J, Kleesiek J. 2024. Cellvit: vision transformers for precise cell segmentation and classification. Med Image Anal 94:103143. doi:10.1016/j.media.2024.103143 38507894

[29] Niazi MKK, Pennell M, Elkins C, Hemminger J, Jin M, Kirby S, Kurt H, Miller B, Plocharczyk E, Roth R, Ziegler R, Shana’ah A, Racke F, Lozanski G, Gurcan MN. 2013. Entropy based quantification of Ki-67 positive cell images and its evaluation by a reader study p 175–183 SPIE. doi:10.1117/12.2007909

[30] Robertson AR. 1990. Historical development of CIE recommended color difference equations. Color Res Appl 15:167–170. doi:10.1002/col.5080150308

[31] Keane TM, Goodstadt L, Danecek P, White MA, Wong K, Yalcin B, Heger A, Agam A, Slater G, Goodson M, et al.. 2011. Mouse genomic variation and its effect on phenotypes and gene regulation. Nature 477:289–294. doi:10.1038/nature10413 21921910 PMC3276836

[32] Specht AG, Kurtz SL, Elkins KL, Specht H, Beamer G. 2022. BCG vaccination of Diversity Outbred mice induces cross-reactive antibodies to SARS-CoV-2 spike protein. bioRxiv. doi:10.1101/2022.04.18.488640

[33] Hastie T, Tibshirani R, Wainwright M. 2015. Statistical learning with sparsity: the lasso and generalizations. CRC press.

[34] Pedregosa F, Varoquaux G, Gramfort A, Michel V, Thirion B, Grisel O, Blondel M, Prettenhofer P, Weiss R, Dubourg V, Vanderplas J, Passos A, Cournapeau D, Brucher M, Perrot M, Duchesnay E. 2011. Scikit-learn: machine learning in Python. J Mach Learn Res 12:2825.

[35] Chen T, Guestrin C. XGBoost: a scalable tree boosting system Proceedings of the 22nd ACM SIGKDD international conference on knowledge discovery and data mining, p 785–794 ACM, San Francisco California USA

[36] Hastie T, Tibshirani R, Friedman J. 2009. The elements of statistical learning: data mining, inference, and prediction. Springer, New York City, USA.

[37] Robin X, Turck N, Hainard A, Tiberti N, Lisacek F, Sanchez J-C, Müller M. 2011. pROC: an open-source package for R and S+ to analyze and compare ROC curves. BMC Bioinformatics 12:77. doi:10.1186/1471-2105-12-77 21414208 PMC3068975

[38] Mason SJ, Graham NE. 2002. Areas beneath the relative operating characteristics (ROC) and relative operating levels (ROL) curves: statistical significance and interpretation. Quart J Royal Meteoro Soc 128:2145–2166. doi:10.1256/003590002320603584

[39] Seabold S, Perktold J. Statsmodels: econometric and statistical modeling with python 9th Python in Science Conference

[40] Waskom ML. 2021. Seaborn: statistical data visualization. J Open Source Software 6:3021. doi:10.21105/joss.03021

[41] Chen EY, Tan CM, Kou Y, Duan Q, Wang Z, Meirelles GV, Clark NR, Ma’ayan A. 2013. Enrichr: interactive and collaborative HTML5 gene list enrichment analysis tool. BMC Bioinformatics 14:128. doi:10.1186/1471-2105-14-128 23586463 PMC3637064

[42] Tavolara TE, Niazi MKK, Gower AC, Ginese M, Beamer G, Gurcan MN. 2021. Deep learning predicts gene expression as an intermediate data modality to identify susceptibility patterns in Mycobacterium tuberculosis infected Diversity Outbred mice. EBioMedicine 67:103388. doi:10.1016/j.ebiom.2021.103388 34000621 PMC8138606

[43] Jacob JT, Mehta AK, Leonard MK. 2009. Acute forms of tuberculosis in adults. Am J Med 122:12–17. doi:10.1016/j.amjmed.2008.09.018 19114163

[44] Basaraba RJ, Hunter RL. 2017. Pathology of tuberculosis: how the pathology of human tuberculosis informs and directs animal models. Microbiol Spectr 5. doi:10.1128/microbiolspec.TBTB2-0029-2016 PMC11687511 28597826

[45] Hunter RL, Jagannath C, Actor JK. 2007. Pathology of postprimary tuberculosis in humans and mice: contradiction of long-held beliefs. Tuberculosis (Edinb) 87:267–278. doi:10.1016/j.tube.2006.11.003 17369095

[46] Leong F-M, Eum S, Via LE, Barry III CE. 2011. Pathology of tuberculosis in the human lung, p 53–81. In Leong FJ, Dartois V, Dick T (ed), A color Atlas of comparative pathology of pulmonary tuberculosis. CRC Press, New York.

[47] Mustafa T, Phyu S, Nilsen R, Jonsson R, Bjune G. 1999. A mouse model for slowly progressive primary tuberculosis. Scand J Immunol 50:127–136. doi:10.1046/j.1365-3083.1999.00596.x 10447916

[48] Kassa GM, Tadesse A, Gelaw YA, Alemayehu TT, Tsegaye AT, Tamirat KS, Akalu TY. 2020. Predictors of mortality among multidrug-resistant tuberculosis patients in central Ethiopia: a retrospective follow-up study. Epidemiol Infect 148:e258. doi:10.1017/S0950268820002514 33054897 PMC7689597

[49] Tiemersma EW, van der Werf MJ, Borgdorff MW, Williams BG, Nagelkerke NJD. 2011. Natural history of tuberculosis: duration and fatality of untreated pulmonary tuberculosis in HIV negative patients: a systematic review. PLoS One 6:e17601. doi:10.1371/journal.pone.0017601 21483732 PMC3070694

[50] Dodd CE, Schlesinger LS. 2017. New concepts in understanding latent tuberculosis. Curr Opin Infect Dis 30:316–321. doi:10.1097/QCO.0000000000000367 28177961

[51] Behr MA, Edelstein PH, Ramakrishnan L. 2018. Revisiting the timetable of tuberculosis. BMJ 362:k2738. doi:10.1136/bmj.k2738 30139910 PMC6105930

[52] Scott NR, Swanson RV, Al-Hammadi N, Domingo-Gonzalez R, Rangel-Moreno J, Kriel BA, Bucsan AN, Das S, Ahmed M, Mehra S, Treerat P, Cruz-Lagunas A, Jimenez-Alvarez L, Muñoz-Torrico M, Bobadilla-Lozoya K, Vogl T, Walzl G, du Plessis N, Kaushal D, Scriba TJ, Zúñiga J, Khader SA. 2020. S100A8/A9 regulates CD11b expression and neutrophil recruitment during chronic tuberculosis. J Clin Invest 130:3098–3112. doi:10.1172/JCI130546 32134742 PMC7259997

[53] Won EJ, Choi JH, Cho YN, Jin HM, Kee HJ, Park YW, Kwon YS, Kee SJ. 2017. Biomarkers for discrimination between latent tuberculosis infection and active tuberculosis disease. J Infect 74:281–293. doi:10.1016/j.jinf.2016.11.010 27871809

[54] Pan S-J, Tapley A, Adamson J, Little T, Urbanowski M, Cohen K, Pym A, Almeida D, Dorasamy A, Layre E, Young DC, Singh R, Patel VB, Wallengren K, Ndung’u T, Wilson D, Moody DB, Bishai W. 2015. Biomarkers for tuberculosis based on secreted, species-specific, bacterial small molecules. J Infect Dis 212:1827–1834. doi:10.1093/infdis/jiv312 26014799 PMC4633767

[55] Zárate-Bladés CR, Silva CL, Passos GA. 2011. The impact of transcriptomics on the fight against tuberculosis: focus on biomarkers, BCG vaccination, and immunotherapy. Clin Dev Immunol 2011:192630. doi:10.1155/2011/192630 21197423 PMC3010624

[56] Wallis RS, Doherty TM, Onyebujoh P, Vahedi M, Laang H, Olesen O, Parida S, Zumla A. 2009. Biomarkers for tuberculosis disease activity, cure, and relapse. Lancet Infect Dis 9:162–172. doi:10.1016/S1473-3099(09)70042-8 19246020

[57] Pai M, Behr MA, Dowdy D, Dheda K, Divangahi M, Boehme CC, Ginsberg A, Swaminathan S, Spigelman M, Getahun H, Menzies D, Raviglione M. 2016. Tuberculosis. Nat Rev Dis Primers 2:16076. doi:10.1038/nrdp.2016.76 27784885

[58] Rangaka MX, Wilkinson KA, Glynn JR, Ling D, Menzies D, Mwansa-Kambafwile J, Fielding K, Wilkinson RJ, Pai M. 2012. Predictive value of interferon-gamma release assays for incident active tuberculosis: a systematic review and meta-analysis. Lancet Infect Dis 12:45–55. doi:10.1016/S1473-3099(11)70210-9 21846592 PMC3568693

[59] Pinto LM, Grenier J, Schumacher SG, Denkinger CM, Steingart KR, Pai M. 2012. Immunodiagnosis of tuberculosis: state of the art. Med Princ Pract 21:4–13. doi:10.1159/000331583 22024473

[60] Tsegaye Y, Admassu W, Edao A, Kinde S, Gentu M, Negash M, Wondmagegn T, Gize A, Zewdie M, Bobosha K, Wassie L. 2022. Alteration of endocrine hormones and antibody responses in different spectrum of tuberculosis disease. Front Immunol 13:849321. doi:10.3389/fimmu.2022.849321 35281036 PMC8913483

[61] Fischinger S, Cizmeci D, Shin S, Davies L, Grace PS, Sivro A, Yende-Zuma N, Streeck H, Fortune SM, Lauffenburger DA, Naidoo K, Alter G. 2021. A Mycobacterium tuberculosis specific IgG3 signature of recurrent tuberculosis. Front Immunol 12:729186. doi:10.3389/fimmu.2021.729186 34630406 PMC8493041

[62] Lee JY, Kim B-J, Koo H-K, Kim J, Kim J, Kook Y-H, Kim B-J. 2020. Diagnostic potential of IgG and IgA responses to Mycobacterium tuberculosis antigens for discrimination among active tuberculosis, latent tuberculosis infection, and non-infected individuals. Microorganisms 8:979. doi:10.3390/microorganisms8070979 32629849 PMC7409123

[63] Morgan J, Muskat K, Tippalagama R, Sette A, Burel J, Lindestam Arlehamn CS. 2021. Classical CD4 T cells as the cornerstone of antimycobacterial immunity. Immunol Rev 301:10–29. doi:10.1111/imr.12963 33751597 PMC8252593

[64] Esaulova E, Das S, Singh DK, Choreño-Parra JA, Swain A, Arthur L, Rangel-Moreno J, Ahmed M, Singh B, Gupta A, Fernández-López LA, de la Luz Garcia-Hernandez M, Bucsan A, Moodley C, Mehra S, García-Latorre E, Zuniga J, Atkinson J, Kaushal D, Artyomov MN, Khader SA. 2021. The immune landscape in tuberculosis reveals populations linked to disease and latency. Cell Host Microbe 29:165–178. doi:10.1016/j.chom.2020.11.013 33340449 PMC7878437

