# Supplementary material for: B cells in perivascular and peribronchiolar granuloma-associated lymphoid tissue and B-cell signatures identify asymptomatic Mycobacterium tuberculosis lung infection in Diversity Outbred mice
Source: Infect Immun. 2024 Jun 20;92(7):e00263-23. doi: 10.1128/iai.00263-23 (PMC11238564; doi:10.1128/iai.00263-23)
Supplement: Supplemental material — Figures S1 to S6, Tables S1 to S3, and supplemental methods. [file iai.00263-23-s0002.docx]

# **SUPPLEMENTARY FIGURES & TABLES**

| a)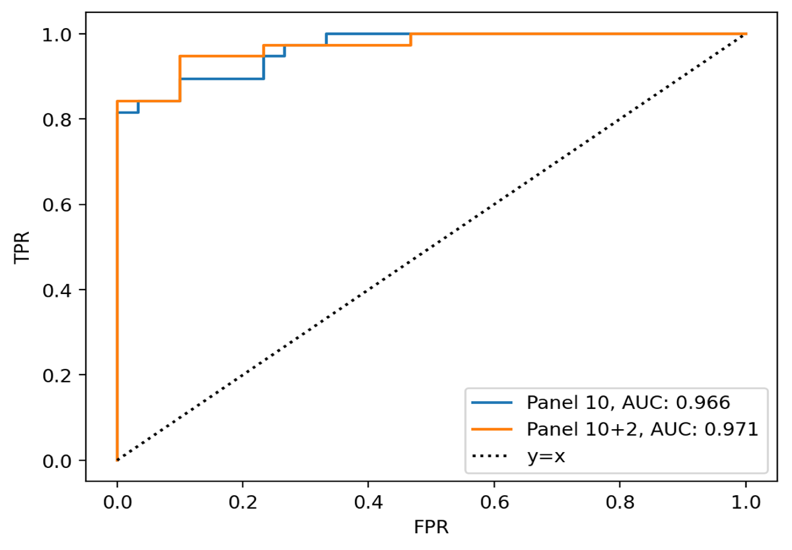 | b)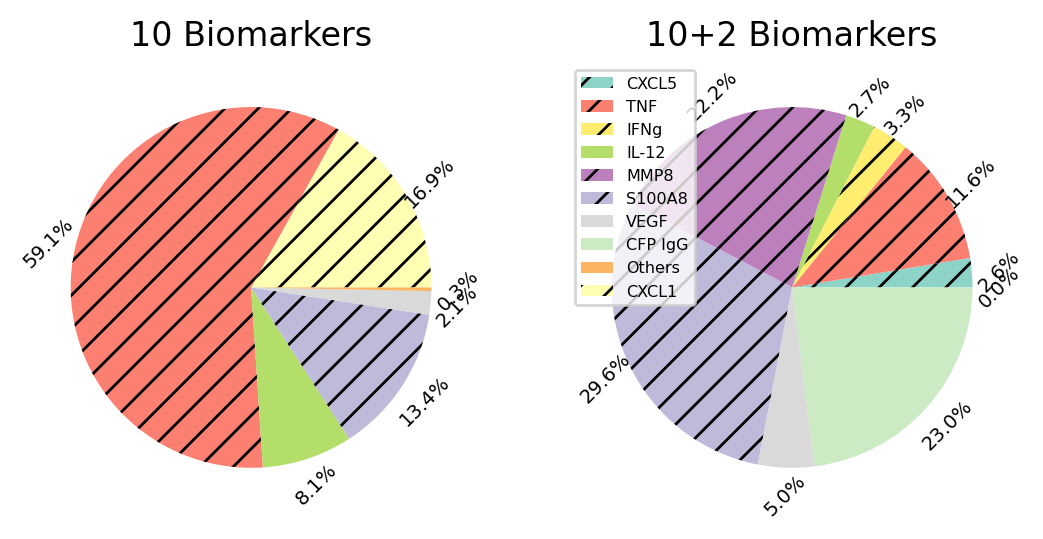 |
| --- | --- |
| **Figure S1** a) ROC curve comparison of the ten-biomarker panel (blue) and twelve-biomarker panel (orange) for the classification between Progressors and Asymptomatic mice. b) Percent Importance of different biomarkers for classifying between Progressors and Asymptomatic mice. Logistic regression is the classifier and importance scores are averaged over 30 folds. Biomarkers corresponding to the unhatched colors are associated with longer survival and vice versa for the hatched colors. Biomarkers with less than 1% importance are omitted. | |

| a) 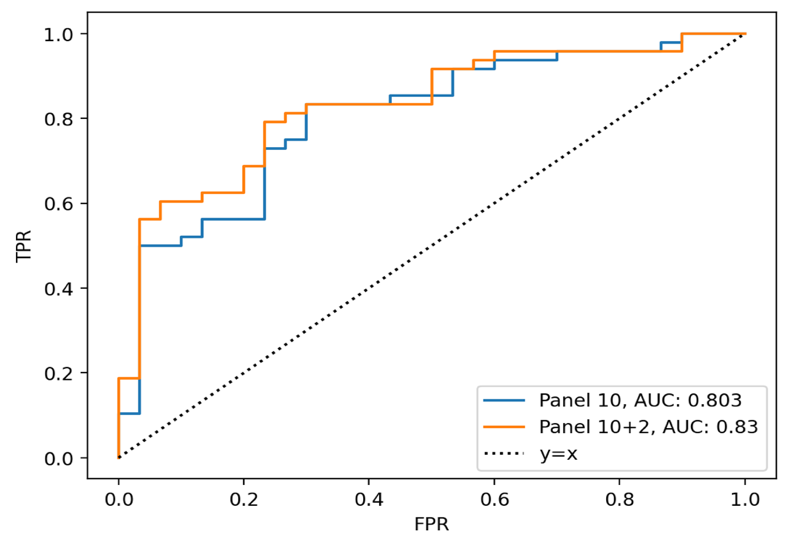 | b)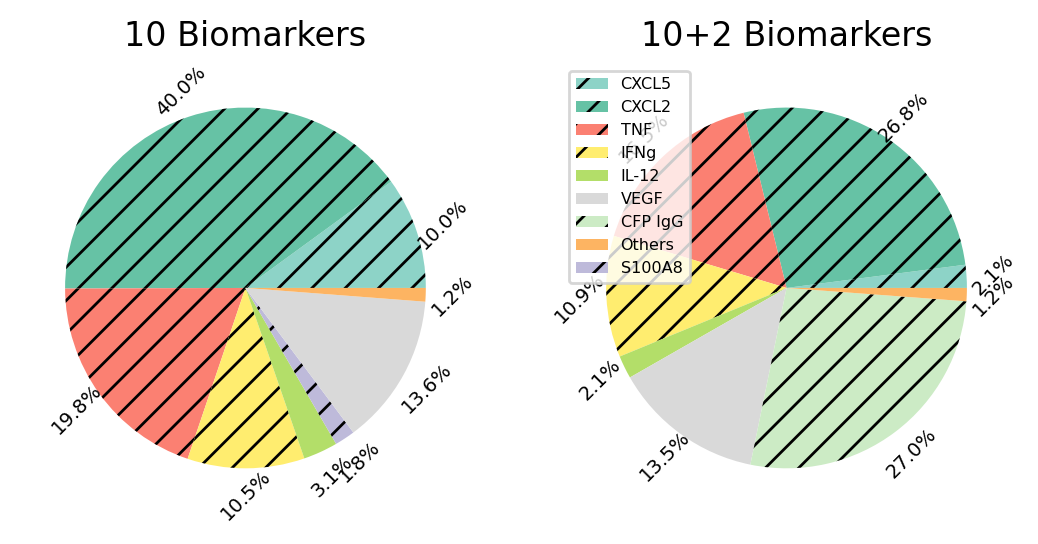 |
| --- | --- |
| **Figure S2**:a) ROC curve comparison of the ten-biomarker panel (blue) and twelve-biomarker panel (orange) for the classification between Controllers and Asymptomatic mice. b) Percent Importance of different biomarkers for classifying between Controllers and Asymptomatic mice. Logistic regression is the classifier and importance scores are averaged over 30 folds. Biomarkers corresponding to the unhatched colors are associated with longer survival and vice versa for the hatched colors. Biomarkers with less than 1% importance are omitted. | |

| a.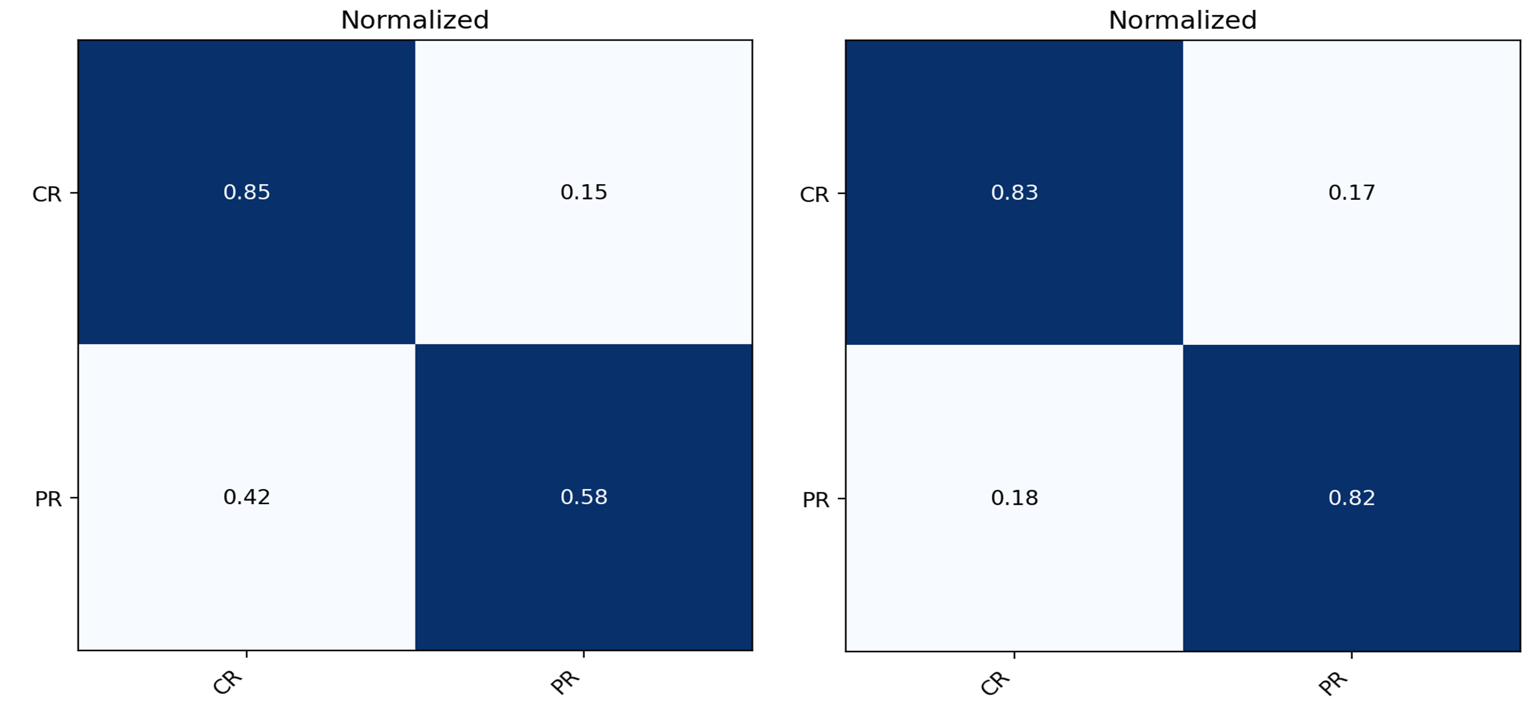 |
| --- |
| b.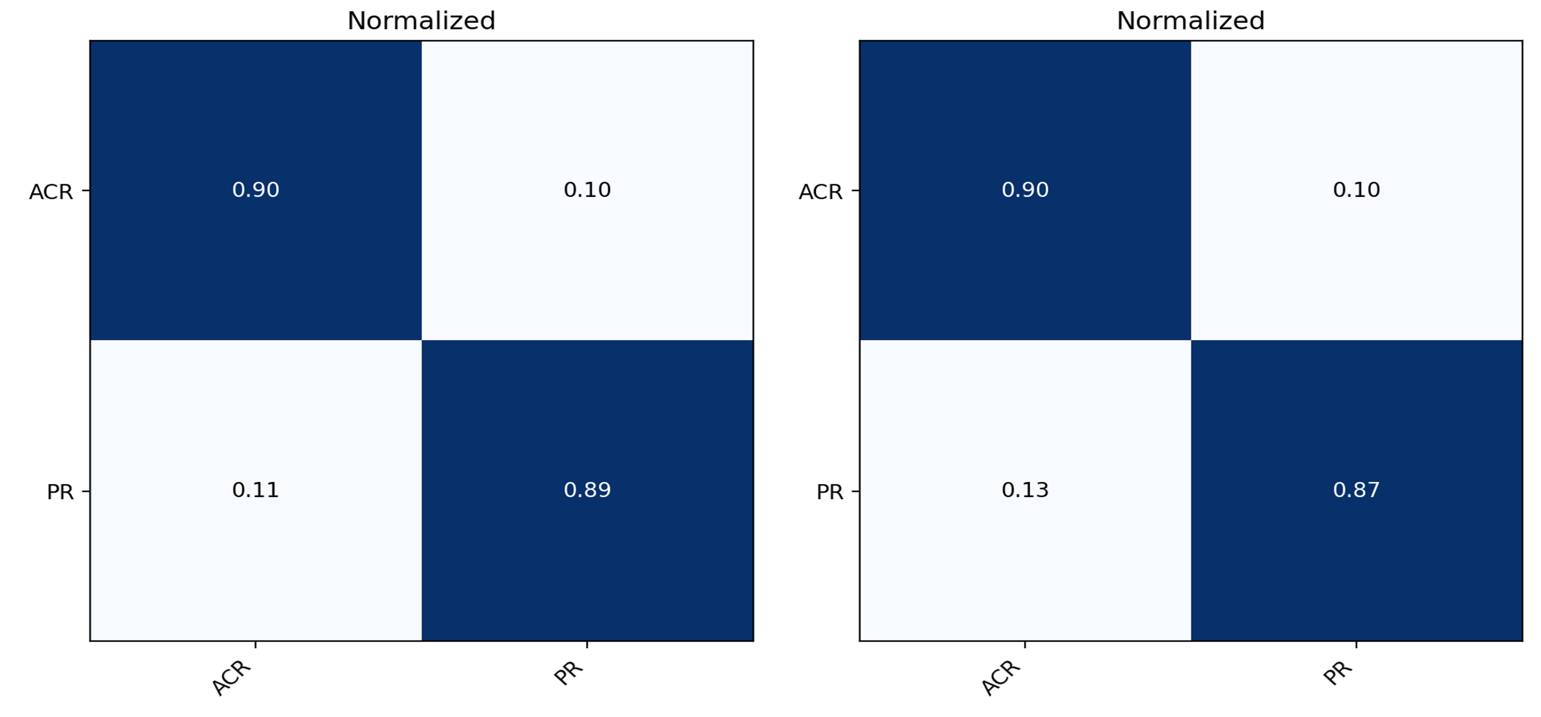 |
| c.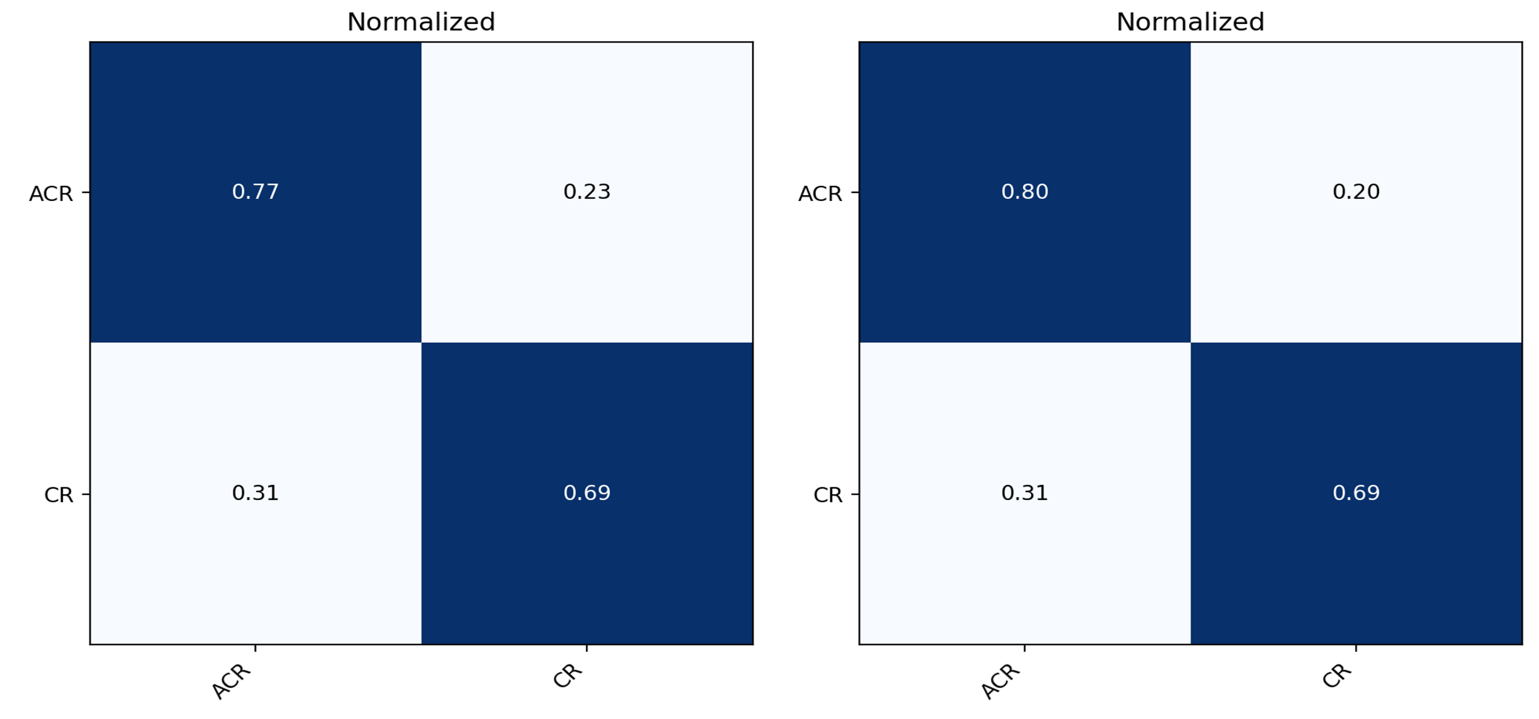 |
| **Figure S3**: Confusion matrices for three different classification tasks: a. Controllers vs Progressors, b. Asymptomatic mice vs Progressors and c. Asymptomatic mice vs Controllers. For each classification task, we display two confusion matrices corresponding to results of panels with 10 (left) and 12 (right) biomarkers respectively. PR: Progressors, CR: Controllers, ACR: Asymptomatic mice. |

| **Table S1.** Performance of the two panels (10 & 10+2 antibodies) in Controllers (CR) vs Asymptomatic mice (AS) using Xgboost. For each comparison best result is highlighted in bold. The positive class is CR. Confusion matrices are given. | | |
| --- | --- | --- |
| *Metric* | *CR vs AS* | |
|  | *10* | *10+2* |
| AUC | 0.77 | **0.774** |
| Sens. (%) | 72.9 | 72.9 |
| Spec. (%) | **80.0** | 76.7 |

| 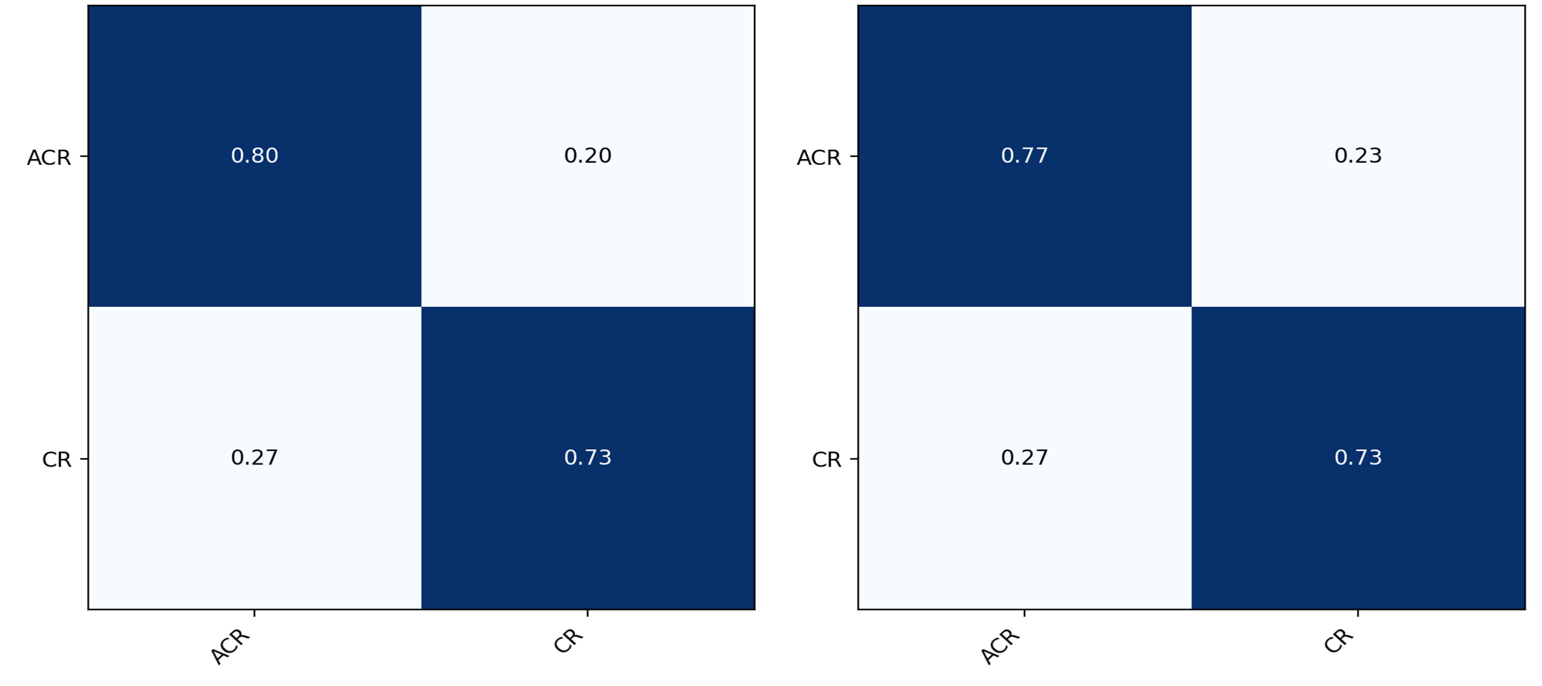 |
| --- |
| **Figure S4: Confusion matrices corresponding to the Xgboost model for Asymptomatic vs Controller comparison. Left and right columns correspond to panels with 10 and 12 biomarkers respectively. CR: Controllers, ACR: Asymptomatic mice.** |

| **Table S2.** Non-infected mice classification performance of the two panels (10 & 10+2 antibodies) trained on three classification problems (Progressors vs Controllers, Progressors vs Asymptomatic mice, and Controllers vs Asymptomatic mice). All n=22 non-infected mice with complete data are used and none of the non-infected mice are used during model training. A prediction is deemed correctly classified if it is predicted as the negative class. Last row denotes the confidence of the model i.e. the average probability of a non-infected mice belonging to the negative class. It indicates the non-infected samples are close to the decision boundary of the panel with the least classification accuracy (31.8%). PR: Progressors, CR: Controllers, AS: Asymptomatic mice, NI: Non-infected mice. | | | | | | |
| --- | --- | --- | --- | --- | --- | --- |
| *Metric* | *Trained for*  *PR vs CR* | | *Trained for*  *PR vs AS* | | *Trained for*  *CR vs AS* | |
|  | *10* | *10+2* | *10* | *10+2* | *10* | *10+2* |
| Accuracy for NI mice (%) | 100 | 31.8 | 95.5 | 90.9 | 90.9 | 90.9 |
| Avg. Prob. (%) | 68 | 45.1 | 84.2 | 70.3 | 62.6 | 65.8 |

| 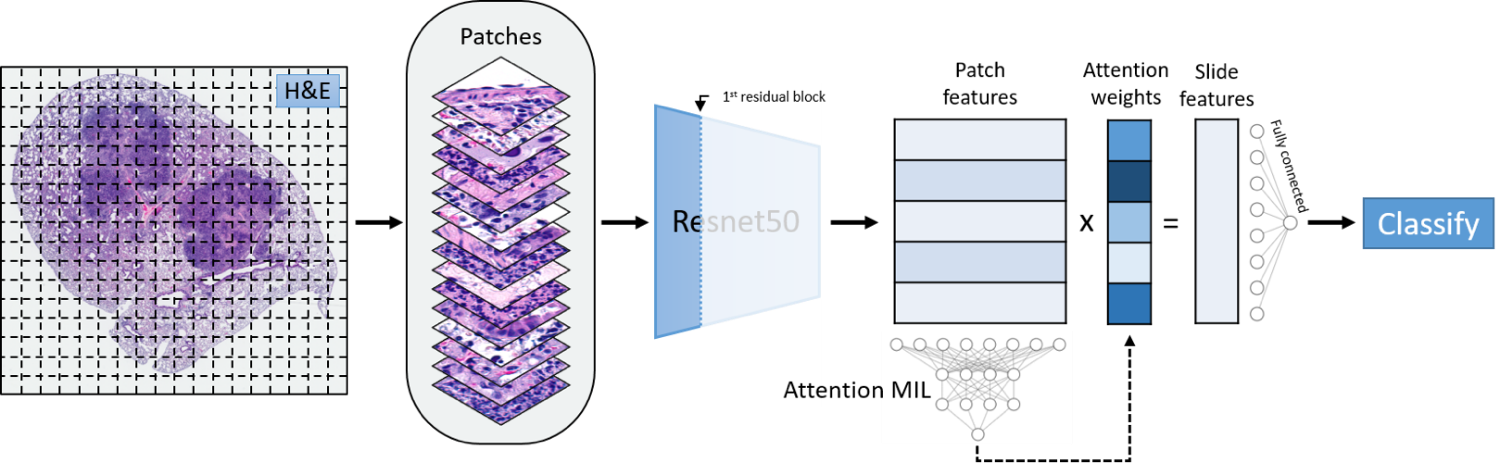 |
| --- |
| **Figure S5** Model overview. Each embedded instance is passed through a shallow feature extractor consisting of two fully connected layers with ReLU activation. Then, each instance is passed through the attention mechanism. This consists of two parallel fully connected layers, a dot product between their outputs, then a final fully connected layer to yield an attention weight. Attention weights are scaled using softmax then dotted with respective outputs from the shallow feature extractor. The resulting slide-level feature vector is then classified into either controller or asymptomatic category. |
| 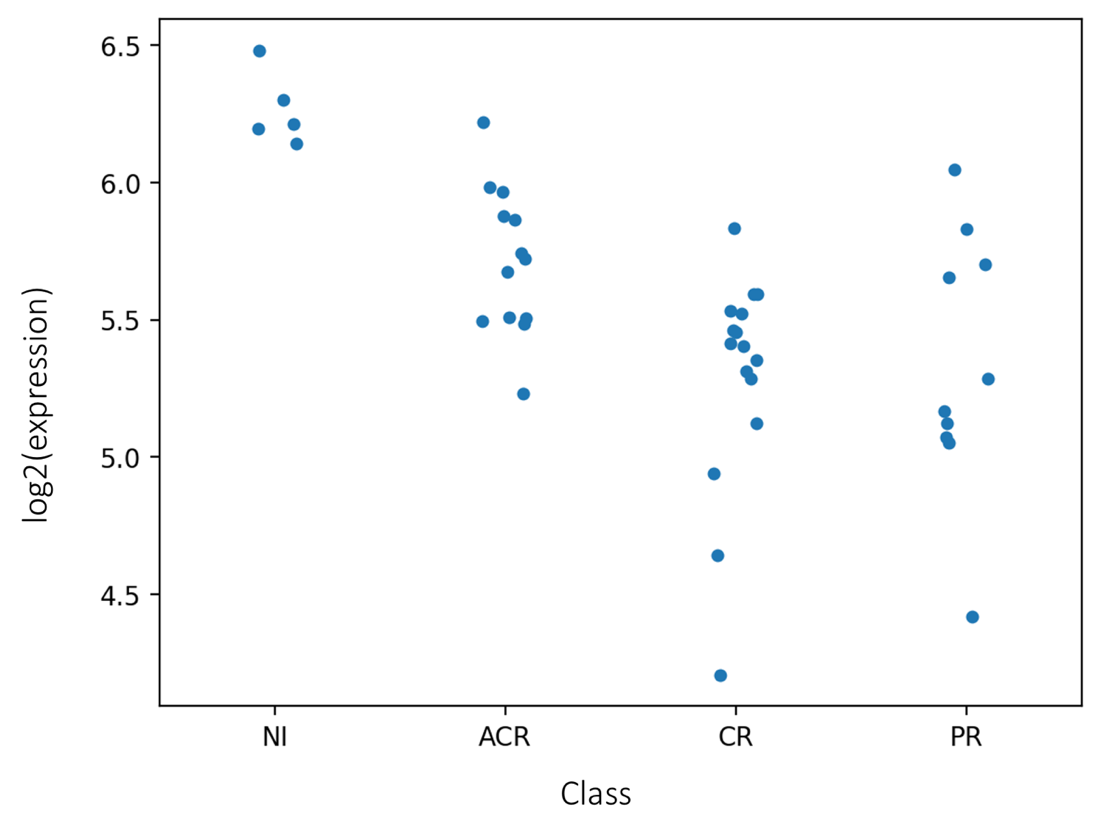 |
| **Figure S6**: Log2 expression of gene F2rl1. Although F2rl1 had high AUC (0.83) for classifying between Asymptomatic and Controller, expression of the gene was lower in asymptomatic mice compared to the non-infected Diversity Outbred mice. NI: non-infected mice, ACR: asymptomatic mice, CR: controllers, PR: progressors. |

| **Table S3.** Microarray Enrichr analysis resulting from the genes selected for the Asymptomatic vs Progressor classification. Adjusted *p*-value <0.05 are displayed. | | | |
| --- | --- | --- | --- |
| **Term** | **Overlap** | **Adjusted P-value** | **Genes** |
| RNA Processing | 46/183 | 0.0005 | ZCCHC8;CELF1;DDX47;CELF2;TUT4;AKAP8L;SRSF1;DDX20;HNRNPR;NOL9;RNPC3;USP39;PRPF8;CCAR2;EXOSC7;ATXN1;RSRC1;EXOSC9;EXOSC8;DHX16;RBM5;RBM6;DDX17;IVNS1ABP;SRPK2;MBNL1;SF3A1;CPSF6;RNGTT;DIS3;NCBP2;METTL3;THOC1;PRPF4B;SRPK1;SETX;PRPF6;SON;DKC1;XRN2;MPHOSPH10;LUC7L3;SNRNP48;HNRNPH3;TARDBP;SRSF7 |
| Regulation Of DNA-templated Transcription Elongation | 17/44 | 0.0052 | CCNT2;LDB1;SRCAP;PARP1;INTS3;INTS10;THOC1;HTATSF1;THOC5;CCAR2;WDR82;INTS4;KAT7;INTS7;ERCC6;INTS9;INTS8 |
| RNA Metabolic Process | 30/111 | 0.0052 | FTO;CELF2;DDX20;PARN;NOL9;EXOSC7;ATXN2;ATXN1;PAPOLG;EXOSC9;DHX36;EXOSC8;PAPOLA;MTREX;RBM5;RBM6;DDX17;RNGTT;DIS3;RNASE6;THOC1;DICER1;SETX;AGO3;DKC1;AGO4;AGO1;XRN2;MPHOSPH10;HNRNPH3 |
| Chromatin Remodeling | 49/228 | 0.0068 | KDM5A;PHF1;CHD8;HP1BP3;CHD6;PSIP1;RSF1;CTCF;CHD3;MYSM1;HPF1;MECOM;DPF2;SMARCAD1;KDM6A;SMARCC1;PBRM1;PCID2;KDM2B;PIH1D1;ATRX;ACTR8;BAZ1B;ARID1A;KAT2A;MTF2;WAC;ASF1A;INO80D;ZBTB1;BAZ2A;PRIMPOL;PAK1;BRD7;MTA2;MTA3;BPTF;SRPK2;PCGF6;CHTOP;KDM4C;BICRAL;GLMN;SMARCA4;MCM3AP;TADA2A;BCOR;ERCC6;EZH1 |
| Nuclear-Transcribed mRNA Catabolic Process | 24/85 | 0.0120 | HBS1L;DDX5;NBAS;CNOT6L;NCBP2;TUT4;PARN;UPF3B;CNOT10;SMG5;ZFP36L1;EXOSC7;CNOT4;PAN2;XRN1;CNOT1;EXOSC9;CNOT2;XRN2;EXOSC8;ATM;EIF3E;DCPS;DIS3L2 |
| DNA Damage Response | 71/384 | 0.0120 | SMARCAL1;WDR48;DYRK2;TRRAP;PHF1;ALKBH8;TNFSF13B;RPTOR;NIPBL;HPF1;HERC2;POLI;KMT5B;MTREX;TRAPPC13;SHPRH;PARP1;RFC2;METTL3;ATRX;RECQL;HUS1;UBE2E2;VRK1;PPP4R3B;BAZ1B;INIP;DDB2;FOXP1;MSH6;NSMCE4A;MSH2;IRF3;RRM2B;WAC;ASF1A;PRKDC;STXBP4;USP10;ZBTB1;PDS5B;CRIP1;FOXO1;NPAS2;PAK1;WRN;OARD1;RBBP5;HLTF;UBQLN4;MAP2K6;MBD4;UVRAG;NEK4;XRCC5;FANCL;INTS3;RPA1;MTOR;SETX;ETAA1;RAD50;TTC5;ERCC4;TDP1;SHLD2;PWWP3A;BCL2;ATM;ATR;TAF1 |
| miRNA Processing | 13/32 | 0.0120 | DDX5;NCBP2;TUT4;SRRT;METTL3;DICER1;PUM1;AGO3;AGO4;PRKRA;AGO1;DGCR8;SRSF3 |
| mRNA Destabilization | 15/43 | 0.0210 | FTO;ROCK1;CELF1;TUT4;METTL3;PUM1;ZFP36L1;AGO3;AGO4;CNOT1;DHX36;METTL16;TARDBP;DCPS;DIS3L2 |
| mRNA Metabolic Process | 23/85 | 0.0219 | DDX5;SF3A1;CPSF6;SSB;CELF1;NCBP2;CPSF3;AKAP8L;METTL3;SRSF1;HNRNPR;CNOT10;USP39;NUDT16L1;PRPF8;SND1;SON;AGO3;AGO4;HSF1;METTL16;SRSF7;DIS3L2 |
| Chromatin Organization | 52/268 | 0.0273 | KDM5A;HDAC10;PHF1;CHD8;CHD6;EHMT1;PSIP1;RSF1;CTCF;CHD3;MYSM1;CABIN1;ING4;DPF2;SMARCAD1;KAT7;HMG20A;HMGN3;KDM6A;SMARCC1;PBRM1;KDM2B;PIH1D1;ATRX;ACTR8;BAZ1B;ARID1A;KAT2A;MTF2;WAC;DNAJC9;ASF1A;INO80D;ZBTB1;BAZ2A;HDAC8;PAK1;BRD7;MTA2;MTA3;BPTF;PCGF6;CHTOP;KDM4C;BICRAL;SMARCA4;CDAN1;TADA2A;PWWP3A;BCOR;ERCC6;EZH1 |
| Regulation Of TORC1 Signaling | 16/50 | 0.0273 | SZT2;PIH1D1;NPRL2;NPRL3;CLEC16A;GPR137B;RNF167;SESN3;NPC1;RRAGB;CASTOR2;RRAGD;WAC;SLC38A9;ATM;DEPDC5 |
| miRNA Metabolic Process | 8/15 | 0.0273 | DDX17;DDX5;TUT4;XPO5;PARN;DICER1;SND1;DIS3L2 |
| mRNA Splicing, Via Spliceosome | 43/211 | 0.0278 | ZCCHC8;DDX5;DDX46;SRSF1;DDX20;HNRNPR;WDR83;HTATSF1;RNPC3;PRPF19;USP39;PRPF8;PQBP1;COIL;EFTUD2;PNN;RSRC1;DHX16;MTREX;DCPS;RBM10;RBM5;RBM6;DDX17;SRRM2;SRPK2;PRMT5;AQR;SF3A1;IK;NCBP2;METTL3;CDC5L;PRPF4B;SRPK1;PRPF6;CWF19L1;SRSF3;HNRNPH3;GEMIN7;SNRNP200;SRSF7;FRA10AC1 |
| Cellular Response To Leucine Starvation | 7/12 | 0.0316 | RNF167;SESN3;RRAGB;RRAGD;EIF2AK4;GCN1;MTOR |
| DNA Metabolic Process | 54/288 | 0.0316 | SMARCAL1;TOP2B;TRRAP;HPF1;SMARCAD1;POLI;KMT5B;TOP3B;PARP1;RFC2;ATRX;RECQL;HUS1;ACTR8;GLRX2;TERF1;INIP;DDB2;MSH6;NSMCE4A;MSH2;RRM2B;TERF2IP;NEIL1;DNASE1L3;ASF1A;EXD2;INO80D;PARG;PRKDC;ZBTB1;PDS5B;WRN;ORC3;HLTF;ORC2;POLD2;CTC1;MBD4;UVRAG;XRCC5;FANCL;INTS3;CNBP;RPA1;NOC3L;RAD52;RAD50;ERCC4;TDP1;PWWP3A;UBE2N;ATM;ATR |
| Cellular Response To Amino Acid Starvation | 14/42 | 0.0316 | SZT2;NPRL2;NPRL3;EIF2AK3;EIF2AK4;MTOR;RNF167;SESN3;RRAGB;RRAGD;MAPK1;DEPDC5;GCN1;MAP3K5 |
| Golgi To Plasma Membrane Transport | 15/47 | 0.0316 | BBS2;OSBPL5;EXOC7;GOLPH3L;ANK3;GGA2;ARFRP1;GOLGA4;PREPL;GGA3;RABEP1;EXOC4;EXOC6;EXOC2;VPS35L |
| mRNA Catabolic Process | 14/43 | 0.0391 | DDX5;NBAS;TUT4;METTL3;CNOT10;NUDT16L1;SND1;AGO3;XRN1;AGO4;EXOSC9;XRN2;METTL16;DIS3L2 |
| snRNA Metabolic Process | 9/21 | 0.0487 | ZCCHC8;INTS3;SNAPC3;INTS10;INTS4;INTS7;MTREX;INTS9;INTS8 |
| Golgi Organization | 29/130 | 0.0487 | USP6NL;CUL7;UBXN2B;ATL3;FAM174B;TRAPPC12;TRAPPC11;DYM;CLASP1;CLASP2;RAB2B;PRMT5;COG7;HACE1;COG6;COG5;GBF1;GOLPH3L;COG1;VPS13B;VRK1;TRAPPC8;RAB30;GORASP2;ZW10;GOLGB1;GORASP1;TRIP11;BET1 |
| mRNA Processing | 42/214 | 0.0487 | ZCCHC8;DDX5;DDX46;CELF1;SRSF1;HNRNPR;WDR83;HTATSF1;RNPC3;PRPF19;USP39;PRPF8;EFTUD2;PNN;RSRC1;PAPOLA;DHX16;MTREX;RBM10;RBM5;RBM6;SRRM2;AQR;SF3A1;IK;CPSF6;RNGTT;NCBP2;CPSF3;METTL3;CDC5L;PRPF4B;RPRD2;PRPF6;SON;CWF19L1;SRSF3;HNRNPH3;GEMIN7;SNRNP200;SRSF7;FRA10AC1 |

# **SUPPLEMENTARY METHODS**

**Preprocessing of H&E images**

Prior to model development, images were preprocessed as described in (23) to detect image foreground by thresholding the red channel with a value of 230; filling holes; and removing artifacts by applying an image erosion and dilation with a disk structuring element with a radius of 3. Then, image patches sized 32x32 pixels at 20x magnification were passed through ResNet50 pretrained on ImageNet until the 2nd residual block. The resultant 8x8x256 feature block was collapsed into a 1x256 feature vector (later called embedded instances) by averaging along the first two dimensions.

**Attention-based multiple instance learning**

Attention-based automatically learns to weight embedded instances into a bag-level feature vector that can be subsequently classified. In a single image, we sampled patches (instances) from the lung section image and performed automated feature extraction to generate the instance embeddings. Each embedded instance was weighted, summed, and combined into a single, image-level embedding to allow classification.

$$a_{k}=\frac{exp\left\{ w^{T}\left( tanh\left( Vh_{k}^{T} \right)\cdot sigm\left( Uh_{k}^{T} \right) \right) \right\}}{\sum_{j=1}^{K} exp\left\{ w^{T}\left( tanh\left( Vh_{k}^{T} \right)\cdot sigm\left( Uh_{k}^{T} \right) \right) \right\}}$$

$$z=\sum_{k=1}^{K} a_{k}h_{k}$$

The attention mechanism implementation consists of a simple two-layer fully connected network which passes each instance embedding (hk) though two parallel layers of the network (V,U), applies tanh and sigmoid activation functions to respective outputs, dots the two results, then passes the activation though the second layer (wT), which maps the vector into a single value, its attention weight (ak). The weighted sum of each embedded instance and its attention weight yields a bag-level instance (z). The parameters (V,U,w) for this for this neural network are automatically learned through training of the model.

In addition to performing better than instance-based and embedding-based max and mean pooling approaches, the resulting instance weights allow the model to be interpretable in that the relative magnitudes of instance weights directly correspond to the instance's relative contribution to the overall classification of the bag.

**Further details regarding the classifiers used with lung cytokines, chemokines, and IgG antibodies**

We have used one linear classifier, l1-regularized logistic regression, and one non-linear classifier XGBoost. Logistic regression model parametrizes the conditional distribution of the class labels using a linear combination of the input features, $x\in R^{p},$ i.e. $P\left( Y=1\vee X=x,\beta,b \right)=\left( 1+exp\left( -\beta^{T}x-b \right) \right)^{-1}$. Let $x^{\left( i \right)}$ denote the input features and $y^{\left( i \right)}\in\left\{ -1,1 \right\}$ the corresponding label of the i^th^ sample. Given N independent and identically distributed samples ${\left\{ x^{\left( i \right)},y^{\left( i \right)} \right\}^{N}}_{i=1}$ and the regularization amount $\lambda,$ to estimate the parameters the sum of the negative log-likelihood and $l_{1}$norm of the weights is minimized i.e. $\sum log\left( 1+e^{-y^{\left( i \right)}\left( \beta^{T}x^{\left( i \right)}+b \right)} \right)+\lambda\left\| \beta\right\|_{1}$. XGBoost like gradient tree boosting, (36) is a method for iteratively fitting a set of decision trees whose additive combination determines the predicted class probabilities. At each iteration, the first and second derivatives of the resulting loss function from the existing set of trees guide the selection of the new tree (35). This process is repeated till the desired number of trees is reached.

**Comparison between Mann–Whitney U-statistic and Welch’s t-test**

In this work, motivated by identifying diagnostically powerful genes, we have preferred AUC analysis of the gene expression profiles to a more standard approach such as the t-test. To measure the sensitivity of the selected genes to the statistical test used we have contrasted the selections of the one-sided Mann–Whitney U-statistic with those of one-sided Welch’s t-test. For the comparison between Asymptomatic and all remaining mice (Controllers, Progressors, and non-infected controls) within the Experiment 1, one-sided Welch’s t-test identified 1133 genes that are expressed at significantly (FDR q < 0.05) as opposed to 105 genes identified using the one-sided Mann–Whitney U-statistic. The resulting 1133 selections from Welch’s t-test contained all 105 selections resulting from the Mann–Whitney U-statistic. However, using the Welch’s t-test resulted in genes with diagnostically less powerful to be selected. The gene with the least diagnostic potential had 0.630 AUC in the set resulting from Welch’s t-test as opposed to 0.844 AUC in the set resulting from Mann–Whitney U-statistic (Supplementary File 1). When 1133 selections from Welch’s t-test input to Enrichr, 18 statistically significant (adjusted *p* < 0.05) GO terms were identified and none of them overlapped with 8 GO terms identified with Mann–Whitney U-statistic (Supplementary File 1). Even if selections from the Welch’s t-test contain the previous 105 selections, the order of magnitude higher number of input genes to Enrichr might have decreased the statistical significance of the previously identified pathways.

REFERENCES

35. Chen T, Guestrin C. XGBoost: a scalable tree boosting system Proceedings of the 22nd ACM SIGKDD international conference on knowledge discovery and data mining, p 785–794ACM, San Francisco California USA.

36. Hastie T, Tibshirani R, Friedman J. 2009. The elements of statistical learning: data mining, inference, and prediction. Springer, New York City, USA.

23. Tavolara TE, Niazi MKK, Ginese M, Piedra-Mora C, Gatti DM, Beamer G, Gurcan MN. 2020. Automatic discovery of clinically interpretable imaging biomarkers for Mycobacterium tuberculosis supersusceptibility using deep learning. EBioMedicine 62:103094.
